# Mass Spectrometry Imaging Reveals Abnormalities in Cardiolipin Composition and Distribution in Astrocytoma Tumor Tissues

**DOI:** 10.3390/cancers15102842

**Published:** 2023-05-19

**Authors:** Anna C. Krieger, Luis A. Macias, J. Clay Goodman, Jennifer S. Brodbelt, Livia S. Eberlin

**Affiliations:** 1 Department of Chemistry, The University of Texas at Austin, Austin, TX 78712, USA; 2 Departments of Pathology & Immunology and Neurology, Baylor College of Medicine, Houston, TX 77030, USA; 3 Department of Surgery, Baylor College of Medicine, Houston, TX 77030, USA

**Keywords:** mass spectrometry imaging, cardiolipin, mitochondria, glioma, tumor heterogeneity

## Abstract

**Simple Summary:**

Cardiolipin is an important mitochondrial lipid for organelle and cellular energy production. Cardiolipin has been found in altered abundance and diversity in glioma xenograft models. Here, we utilize mass spectrometry imaging to map cardiolipin alterations in human normal and astrocytoma tumors in the histologically diverse tumor microenvironment. Longer chain cardiolipin species were detected at significantly lower relative abundance in tumor tissues versus normal cortex. Cardiolipin diversity correlated with histological trends in the tumor microenvironment including tumor cell invasion and tumor viability. The expression level of proteins involved in mitochondrial energy production was found to decrease with increasing tumor grade among human glioma tumors, though the major enzyme involved in cardiolipin synthesis was not found to be differentially expressed. This work provides confirmation of cardiolipin alterations in human astrocytomas and provides rationale for a spatially aware approach when considering cardiolipin diversity in the human tumor microenvironment.

**Abstract:**

Cardiolipin (CL) is a mitochondrial lipid with diverse roles in cellular respiration, signaling, and organelle membrane structure. CL content and composition are essential for proper mitochondrial function. Deranged mitochondrial energy production and signaling are key components of glial cell cancers and altered CL molecular species have been observed in mouse brain glial cell xenograft tumors. The objective of this study was to describe CL structural diversity trends in human astrocytoma tumors of varying grades and correlate these trends with histological regions within the heterogeneous astrocytoma microenvironment. To this aim, we applied desorption electrospray ionization coupled with high field asymmetric ion mobility mass spectrometry (DESI-FAIMS-MS) to map CL molecular species in human normal cortex (N = 29), lower-grade astrocytoma (N = 19), and glioblastoma (N = 28) tissues. With this platform, we detected 46 CL species and 12 monolysocardiolipin species from normal cortex samples. CL profiles detected from glioblastoma tissues lacked diversity and abundance of longer chain polyunsaturated fatty acid containing CL species when compared to CL detected from normal and lower-grade tumors. CL profiles correlated with trends in tumor viability and tumor infiltration. Structural characterization of the CL species by tandem MS experiments revealed differences in fatty acid and double bond isomer composition among astrocytoma tissues compared with normal cortex and glioblastoma tissues. The GlioVis platform was used to analyze astrocytoma gene expression data from the CGGA dataset. Decreased expression of several mitochondrial respiratory enzyme encoding-genes was observed for higher-grade versus lower-grade tumors, however no significant difference was observed for cardiolipin synthesis enzyme CRLS1.

## 1. Introduction

Gliomas are the most common primary brain tumor, with an age-standardized incidence rate of 6 in 100,000 persons per year [1,2]. Astrocytomas (AST) are the most common histological subtype of gliomas, the most aggressive of which are the glioblastomas (World Health Organization grade 4 astrocytoma, GBM). GBMs account for 80% of glioma cases and have the poorest 5-year survival rates of all primary brain tumors, at approximately 5% [3]. These aggressive tumors are characterized by abnormal energy production via aerobic glycolysis, implicating underlying mitochondrial dysfunction in glioma pathophysiology [4]. Further, GBMs are largely resistant to apoptosis, resulting in part from abnormalities in the intrinsic, mitochondria-dependent, apoptotic pathway. In particular, p53 gene mutations are observed in 30–50% of human gliomas and their presence in malignant glioma is associated with significantly increased chance of recurrence following resection [4]. Thus, advancing understanding of mitochondrial and energy dysfunction in human gliomas at the molecular level is critical to deepening disease knowledge and assisting development of targeted therapeutics.

Cardiolipins (1,3-diphosphatidyl-*sn*-glycerol, CL) are a mitochondria-specific class of anionic phospholipids that are essential for proper organelle function [4,5]. Predominantly localized to the inner mitochondrial membrane, CLs have a distinctive chemical structure comprised of four acyl chains within two phosphatidylglycerols bridged by a glycerol backbone with two negative charges from the phosphate groups. CL acyl chain diversity, related to the number of carbons, double bonds, and double bond position in each acyl chain, is cell- and tissue-type dependent, suggesting a functional or energetic requirement for such diversity [5,6]. CLs have been increasingly investigated due to their diverse and important roles related to mitochondrial function including electron transport, mitochondrial membrane structure and function, and apoptotic signaling, with direct implications in cancer metabolism and therapeutic approaches [7].

Aberration in CL molecular diversity has been associated with numerous pathologies [5,8,9], and has been increasingly investigated for its role in cancer [7,10,11,12,13,14,15,16]. Changes in CL content in cancer tissues are commonly studied using high-performance liquid chromatography mass spectrometry (HPLC-MS). HPLC-MS has been used to characterize CL species in human non-small cell lung carcinoma cells [12], prostate cancer cells [10], and hepatocellular carcinoma tissue [13], providing new insights into biological mechanisms involved in cancer development. A decrease in CL content and modification of CL molecular species was observed in glioma-susceptible brain [15] and xenograft glial cell tumors in comparison with normal brain in murine models of brain tumors [14]. In particular, this alteration in CL composition in glioma and tumor-susceptible tissue was associated with a significant reduction in the activity of mitochondrial respiratory chain protein complexes. While these studies provide evidence of the importance of CL in glioma pathophysiology, changes in CL distribution in histologically diverse human glioma tissues have not been extensively studied. Moreover, investigations of CL distribution within the tumor microenvironment could provide deeper understanding of their molecular role in tumor development. Yet, while HPLC-MS provides quantitative lipid analysis from biological samples, tissue homogenization precludes a spatial understanding of lipid profiles within heterogenous tissues.

Mass spectrometry (MS) imaging has been extensively used to map spatially registered lipid profiles in human cancer tissues, allowing identification of various phospholipid species as potential markers of disease state [17,18,19,20]. However, due to the diversity of molecular species and relatively low abundance of CL relative to other classes of lipids in tissues, applications of MS imaging for CL mapping from biological samples have been limited. MS imaging studies aimed at CL analysis have utilized methods to reduce ion signal from more abundant lipid classes to allow effective detection of CL ions [21,22,23] or have studied tissues characterized by high cellular abundance of mitochondria, such as in oncocytic thyroid and kidney tumors [11,24]. However, detection of CL in brain tissues where CL is present with multiple isoforms and at lower abundance compared to other phospholipids has been limited to only a few of the most abundant CL species. Coupling of Desorption Electrospray Ionization-MS (DESI-MS) with a high field asymmetric waveform ion mobility (FAIMS) device has been demonstrated as an effective approach to improve the sensitivity of DESI-MS imaging for detection of multiply charged biomolecules, including CL [25]. In FAIMS, gas phase ions are exposed to a differential electric field prior to mass analysis, allowing only a subset of ions to enter the mass spectrometer [26]. This process results in increased signal to noise (S/N) ratios for specific ions through the reduction in chemical noise. As such, DESI-FAIMS-MS provides a powerful approach to studying the composition and spatial distribution of CL in biological samples. 

Here, we applied DESI-FAIMS-MS imaging to investigate the molecular composition and distribution of CL in human normal cortex (NL), AST 1 and 2 (WHO grades 1 and 2), and GBM tissues. Spatially registered DESI-FAIMS mass spectra and statistical analyses allowed comparison of CL profiles among tumor types and among histological regions within heterogeneous patient samples, enabling identification of significant differences in CL distribution among tissue types. Immunofluorescence staining and confocal microscopy were performed to investigate mitochondrial distribution in human brain tissues. Expression levels of mitochondrial enzymes involved in energy production were compared among AST and GBM. To achieve a better understanding of CL structural changes in AST and GBM samples, we performed electrospray ionization collision-induced dissociation/ultraviolet photodissociation (ESI-CID/UVPD) tandem MS experiments on selected CL species to characterize double bond isomer composition among acyl chains of CL related to tissue pathologies. Collectively, our study shows that CL dysregulation in human gliomas is markedly characterized by a shift in CL content and composition presenting higher relative abundance of immature species. 

## 2. Materials and Methods

### 2.1. Tissue Samples

Banked human tissue samples including 28 human glioblastomas (GBM, astrocytoma WHO grade 4), 19 lower-grade astrocytoma (AST, 10 WHO grade 1, and 9 WHO grade 2) and 29 non-cancerous brain cortex (NL) specimens were obtained from the University of Alabama Brain SPORE via the Cooperative Human Tissue Network and Baylor St. Luke’s Medical Center at the Baylor College of Medicine under approved institutional review board protocols. Isocitrate dehydrogenase 1 (IDH1) mutation statuses as determined by immunohistochemical staining were provided by the tissue source for a subset of tumor specimens. A list of patient sample demographics can be found in Table S1. Samples were stored at −80 °C until sectioning. Tissues were sectioned at 16 um thickness using a CryoStar NX50 cryostat (Thermo Scientific, San Jose, CA, USA) and mounted onto glass slides. Mounted sections were stored at −80 °C until MS imaging. Prior to imaging experiments, slides were dried for ~10 min. Due to the size of the tumor samples obtained and differences in tissue mass requirements for subsequent analyses, we restricted immunofluorescence staining and CL structural characterization analyses to only a single grade of lower-grade AST tumors.

### 2.2. DESI- and DESI-FAIMS-MS Imaging

A DESI 2D^TM^ system (Prosolia Inc., Indianapolis, IN, USA) coupled to a Q Exactive mass spectrometer (Thermo Scientific, San Jose, CA, USA) was used for tissue imaging. Imaging was performed at a spatial resolution of 200 µm using the histologically compatible solvent system acetonitrile/dimethylformamide 1:1 (*v*/*v*) [27]. FAIMS was operated at voltages optimized for CL transmission (See Supplemental Materials & Methods for additional detail). MS analysis was performed in the negative ion mode from *m*/*z* 100–1500 at a resolving power of 70,000. 

### 2.3. Tissue Staining and Pathological Evaluation

Tissue sections subjected to MS imaging experiments were stained using a standard hematoxylin and eosin (H&E) staining protocol. Pathological evaluation was performed by JCG using light microscopy. Regions containing pure normal cells, pure tumor cells, and necrosis were indicated in each tissue section.

### 2.4. Mitochondrial Isolation, Lipid Identification, and Structural Analysis

The Mitochondria Isolation Kit for Tissue (PIERCE, Rockford, IL, USA) was used to isolate mitochondria from human brain tissues. Total lipid extracts were performed from mitochondrial isolates using the Bligh and Dyer method [28]. From lipid extracts, cardiolipin molecular species were identified using high mass accuracy measurements (<5 ppm) and collision-induced dissociation (CID) tandem MS (MS^n^) analysis, performed on a hybrid LTQ-Orbitrap Elite mass spectrometer (Thermo Scientific, San Jose, CA, USA) at 60,000 resolving power. Fragmentation patterns were compared to literature reports and data from Lipid Maps database (www.lipidmaps.org) (accessed on 25 August 2020) to determine molecular identity. FA unsaturation was analyzed via a hybrid CID/UVPD approach as described by Macias et al. [29] on an Orbitrap Fusion Lumos Tribrid mass spectrometer modified with a Coherent Excistar excimer laser to perform 193 nm UVPD in the high-pressure linear ion trap, as previously described [30]. Carbon-carbon double bond location isomer ratios were determined by comparing the summed abundances of diagnostic ions for FA(18:1) Δ9 and Δ11 unsaturated species. CL structures were described using lipid shorthand notation as described by Liebisch et al. [31].

### 2.5. Immunofluorescence and Confocal Microscopy

Fresh frozen tissue sections (serial to specimens that underwent mass spectrometry imaging) were stained for mitochondria using Alexa Fluor 488 conjugated primary human mitochondria monoclonal antibody MAB1273A4 (Millipore Sigma, Burlington, MA, USA), or Alexa Fluor 488 TOMM20 mitochondrial marker (ab205486, Abcam, Cambridge, UK). DAPI was used as a nuclear stain, and phalloidin was used as a counter stain for f-Actin. Immunofluorescence images were acquired from regions of interest (2–3 ROI per patient sample) on a Zeiss LSM880 confocal microscope. Images were imported to ImageJ/FIJI image processing software version 1.53q (https://imagej.nih.gov/ij/, Bethesda, MD, USA) (accessed on 29 November 2020) for area-of-stain analyses. Single channel images for the two mitochondrial stains and DAPI were converted to greyscale. Area, mean grey value, and area fraction were quantified based on a threshold applied universally across all ROI for each channel, to calculate the positively stained area of each ROI. Area fraction of positive mitochondrial stain was normalized to nuclear stain area and compared among tumor grades.

### 2.6. mRNA Expression Levels

The mRNA expression statuses for TOMM20, MT-CO1, MT-CO2, MT-CO3, MT-ATP6, MT-ATP8, TIMM22, TIMM23, and CRLS1 were assessed by curating publicly available patient gene expression datasets accessed via the bioinformatics analysis and visualization platform GlioVis Version 0.20 running R version 3.3.2 (gliovis.bioinfo.cnio.es) (accessed on 20 July 2021). Comparison of expression levels was restricted to the following primary adult tumors from the CGGA dataset [32] containing mRNA microarray data collected on the Agilent Whole Human Genome (Array) platform: astrocytoma, anaplastic astrocytoma, and glioblastoma. Data was comprised of over 300 samples and gene expression was compared among tumor grades. Significant differences in expression levels of the listed genes were identified using post-hoc tests, Tukey’s Honest Significant Differences, or pairwise *t*-tests with Bonferroni correction for multiple comparisons.

### 2.7. Data Processing and Statistical Analysis

Xcalibur.RAW files were converted using FireFly data conversion software (v2.2.00, Prosolia, Inc., Indianapolis, IN, USA) and ion images were visualized in the open-source imaging software package BioMap (v3.8.0.4, Novartis). CL S/N information was extracted from regions of pure normal cortex or pure, viable glioblastoma using MSiReader [33] and François Allain’s Python bindings for MSFileReader (https://github.com/frallain/pymsfilereader) (accessed on 10 July 2020). Data visualization and statistical analyses including principal component analysis (PCA) and non-parametric hypothesis tests were performed in Rstudio using the R language and packages (ggplot2, ggpubr, FactoMineR, and fvizcluster) from the CRAN R language library. Shapiro-Wilk tests were used to determine normality of distributions to inform use of parametric testing.

## 3. Results

### 3.1. CL Profiles Obtained by DESI-FAIMS-MS Differ by Tissue Pathology and IDH1 Status, and within Tumors According to Tumor Cell Density and Viability

In this study we used DESI-FAIMS-MS imaging to investigate the composition and distribution of CL in normal brain and glioma tissues. DESI-FAIMS-MS imaging was used to characterize AST (N = 19), GBM (N = 28) and NL samples (N = 29). DESI-FAIMS mass spectra were comprised by distinct profiles of ions spaced by 0.5 *m*/*z* difference in the mass range *m*/*z* 690–780, putatively attributed to CL species. High mass accuracy measurements as well as MS^2^ and MS^3^ analyses were used to confirm the identity of these doubly charged species as CL and monolysocardiolipin (MLCL, Figures S1 and S2). Table 1 contains selected CL molecular species *m*/*z* values and corresponding structural identification. A complete list of detected CL can be found in Table S2.

This approach allowed detection of 46 different CL molecular species from NL tissues, consistent with the high degree of acyl chain diversity observed in normal mammalian brain tissues [6,16].

**Figure 1:**
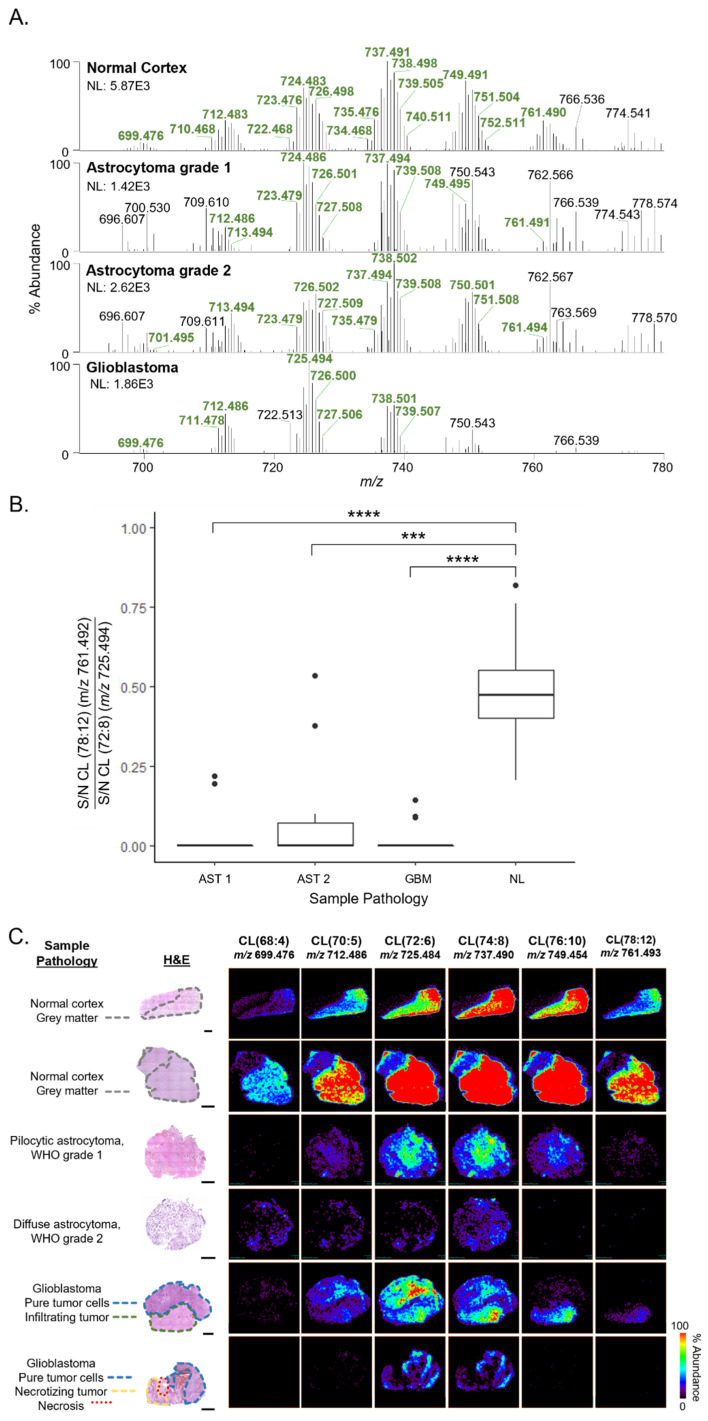
Differences in CL profiles detected by DESI-FAIMS-MS analysis of NL, AST grades 1 and 2, and GBM tissues. (**A**) Representative negative ion mode DESI-FAIMS-MS spectra from *m*/*z* 690–780 from NL, AST, and GBM tissues. Spectra are an average of 10 scans. The *m*/*z* flags of ions corresponding to CL are bolded in green. (**B**) Boxplot comparing S/N of longer chain CL 78:12 (*m*/*z* 761) with shorter chain CL 72:8 (*m*/*z* 725). Wilcoxon tests with Holm adjustment were performed for multiple comparisons. All pairwise comparisons were performed, only significant results are displayed (*** *p* ≤ 0.001, **** *p* ≤ 0.0001). (**C**) DESI-FAIMS-MS ion images of six CL species from NL, AST, and GBM tissues. Optical images of H&E-stained tissues are the same tissue section that was analyzed by MS imaging. “Sample pathology” information indicates tissue pathology and histological regions of interest for each sample. All scale bars are 2 mm.

In contrast to NL tissues, CL profiles detected from AST and GBM tissues presented lower diversity of CL species, where the median number of CL molecular species detected per-sample was fifteen and ten, respectively, compared with an average of twenty-six CL detected in NL samples (Figure 1A). In NL tissues, detected CL molecular species had diverse acyl chains with lengths ranging from sixty-eight to eighty carbons and two to fourteen units of unsaturation among the four acyl chains. As seen in the CL profiles shown in Figure 1A, a distinct pattern largely consisting of six clusters of ions that differ by acyl chain length was detected. Within each cluster, CL molecular species differed by acyl chain unsaturation. For example, prominent molecular species detected in the cluster centered around *m*/*z* 724 had 72 carbons with varying numbers of double bonds, including CL(72:8) (*m*/*z* 723.480), CL(72:7) (*m*/*z* 724.483), CL(72:6) (*m*/*z* 725.494), and CL(72:5) (*m*/*z* 726.503). In NL tissues, the relative abundance of molecular species within each grouping was largely symmetrical, with the most abundant species at the center of the cluster. The most abundant cluster of CL species was centered around *m*/*z* 737.491 (CL(74:8)), and the clusters on either side were detected at a lower relative abundance in a similarly symmetrical pattern as within-cluster patterns. CL profiles of AST tissues were detected at a lower overall abundance with a generally similar distribution of molecular species within each cluster. Notably, CL profiles detected in GBM tissues were characterized by a decrease in abundance of longer acyl chain CL compared with those in NL tissues, such as CL(76:10) (*m*/*z* 749.495) and CL(78:12) (*m*/*z* 761.493). For example, a longer-chain CL species, CL (78:12) (*m*/*z* 761.492), was detected in NL tissues (35%) but not detected in GBM (0%). The cluster of CL with the highest relative abundance in GBM tissues was centered around a shorter chain CL species, CL (72:6) (*m*/*z* 725.494). The S/N of a longer chain and shorter chain CL were compared (CL 78:12 versus the shorter chain CL 72:8), and an average ratio of short/long chain of 0.48 was obtained for NL tissues, with the ratio decreasing significantly to 0.05, 0.10, and 0.01 for AST grade 1, AST grade 2, and GBMs, respectively (all *p* < 0.001, Wilcoxon) (Figure 1B).

**Figure 2:**
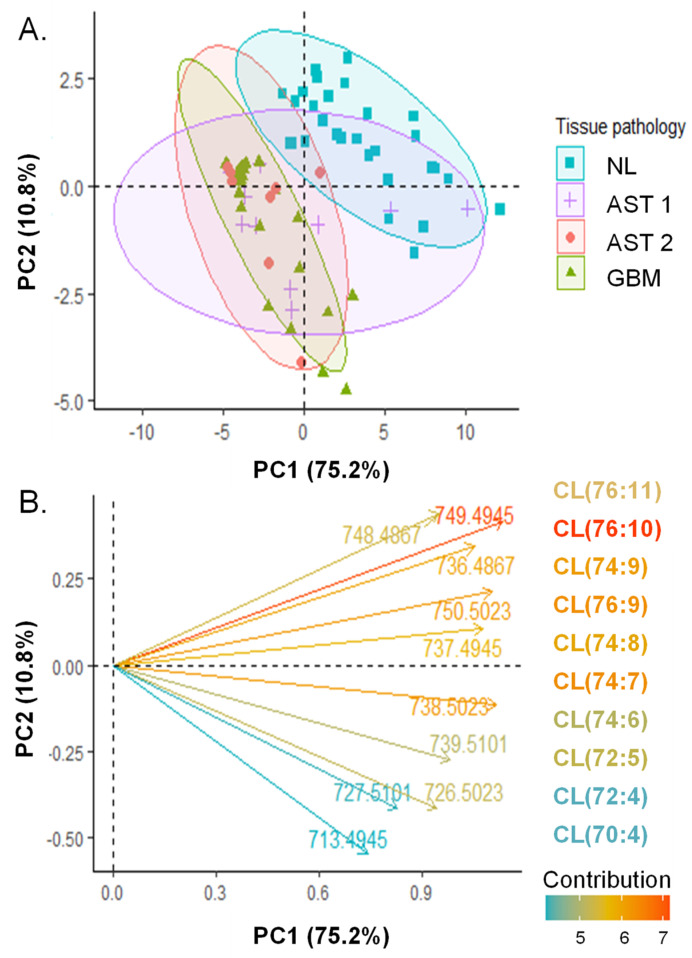
Principal component analysis of CL profiles extracted from regions of pure tumor cell or normal grey matter analyzed by DESI-FAIMS-MS. (**A**) Score plot of individuals. S/N of CL species were extracted from DESI-FAIMS-MS pixels corresponding to regions of pure, viable tumor or normal grey matter and averaged on a per-patient basis. Data are centered and log transformed. PC1 and PC2 account for 75.2% and 10.8% of dataset variability, respectively. 90% concentration ellipses are shown. (**B**) Loading plot showing the direction of contribution of the 10 *m*/*z* features with the highest contribution to the PCs. Feature vectors and their corresponding molecular species are colored according to their contribution (%).

The polyunsaturated, longer acyl chain CL species were found to contribute to the separation of NL and GBM CL profiles by PCA. The median S/N of each CL species detected is shown in Figure S3. PCA was performed on the log-transformed [34], per-patient median S/N of CL species (Figure 2). PCs 1 and 2 explained 72.5% and 10.8% of CL profile variance, respectively. Separation was observed among CL profiles of GBM and NL tissues, while overlap was observed among AST and NL profiles as well as AST and GBM profiles. Among AST samples, no separation was observed between grades 1 and 2 AST profiles. Similar separation patterns are observed when visualizing 95% confidence ellipses, shown in Figure S4. The loadings of CL features were investigated to determine CL species that are important to profile differences among tissue pathologies. The loading plot of the 10 CL molecular species with the greatest contributions to PCs 1 and 2 is shown in Figure 2B. Features with positive contributions to PCs 1 and 2 were *m*/*z* 748.487 (CL(76:11)), *m*/*z* 749.494 (CL(76:11)) and *m*/*z* 736.487 (CL(74:9)) and contributed to separation between NL and GBM CL profiles. Features with positive contributions to PC1 but negative contributions to PC2 contributed to separation within histological groups, and included *m*/*z* 713.495 (CL(70:4)), *m*/*z* 727. 510 (CL(72:4)), and *m*/*z* 726.502 (CL(72:5)). Generally, longer chain and more highly unsaturated CL species contributed to separation between histological groups and shorter chain saturated CL species contributed to variability within histological groups. Further, we obtained preliminary evidence that differences in CL profiles may exist between IDH1 mutant and wild type tumors, warranting additional investigation. PCA was performed on CL S/N values for a subset of 18 specimens with known IDH1 mutation statuses (N = 5 mutant, Mut and N = 11 wild-type, WT). While separation of 90% confidence intervals was not observed (Figure S5A), IDH1 mutant CL profiles clustered along PC2 with major contributions above 20% from *m*/*z* features 723.480 (CL(72:8)) and 724.483 (CL(72:7)) (Figure S5B). The median S/N of these two shorter-chain CL species was compared for viable tumor regions of IDH1 mutant specimens and found to be detected at significantly lower abundance in IDH1 mutant tissues versus IDH1 wild-type tissues (Wilcoxon, P = 0.0275 for *m*/*z* 723.480 (CL(72:8)) and P = 0.0275 *m*/*z* 724.483 (CL(72:7)), Figure S5B,C).

Notably, our study allowed characterization of CL profiles from regions of differing morphologies within the diverse glioma microenvironment, including infiltrating tumor and necrosis. The tumor microenvironment of AST tissues, and particularly GBM, is known to present a high degree of spatial heterogeneity [35,36]. As such, we were interested in characterizing CL profiles in regions of pure tumor, infiltrating tumor, and necrosis. Figure 1C shows DESI-FAIMS-MS ion images of selected CL species for a NL, AST grade 1, AST grade 2, and two GBM sections, as well as optical images of the H&E-stained tissue sections annotated with relevant histological regions. Pathological evaluation revealed regions of cytological atypia and increased nuclear density in AST samples, as well as cytological and nuclear atypia, regions of necrosis, and greatly increased nuclear density in GBM samples. In NL samples, the distribution of CL was colocalized with regions of grey matter. CL signal was not detectable in regions of pure white matter, likely due to ion suppression from abundant myelin-related lipids [16]. Similar to CL profile trends observed from representative mass spectra, CL ion images from tumor samples show decreased overall CL intensity compared with NL tissues, and CL ion images of GBM tissues show a lack of longer chain CL species.

In tumor samples, CL profiles differed among regions of different histology within the same patient tissue (Figure 1B, row 5). For example, histopathological analysis of patient sample 71 identified two distinct histological regions, one containing pure tumor cells satisfying morphological requirements for GBM diagnosis, and the other containing tumor cells diffusely invading into nearby cortex. Comparison of the CL profiles from these two distinct histological regions (Figure S6A) revealed a higher relative abundance of longer chain CL species in regions of infiltrating tumor compared to regions containing pure tumor cells. For example, ion signals for CL(72:6) (*m*/*z* 725.484) displayed a homogenous molecular distribution across both pure and infiltrating tumor regions within the sample, whereas the distribution of a longer chain species with a higher degree of unsaturation, CL(78:12) (*m*/*z* 761.493), was colocalized with regions of infiltrating tumor and the ion was not detected in the pure tumor region. In addition to diffuse invasion of tumor cells, GBM is also characterized by regions of necrosis. Patient sample 60 (Figure 1B, row 6) had regions of viable tumor, tumor in the process of becoming necrotic, and necrosis. Comparison of CL profiles from regions of varying tumor viability (Figure S6B) from this patient sample revealed CL species at greater relative abundance in viable tumor regions (100%) when compared to necrosis (20%). 

### 3.2. MS^n^ Analysis of CL Reveals Differences in CL Molecular Structure among NL, AST, and GBM Tissues

**Figure 3:**
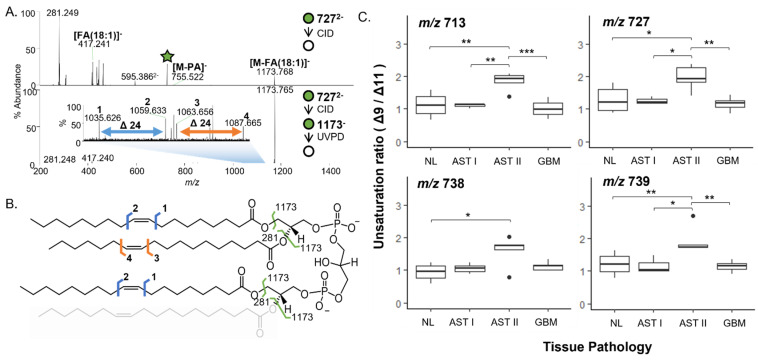
CID-UVPD-MS analysis of double bond isomer ratios. (**A**) CID (**top**) and CID-UVPD (**bottom**) mass spectra from MS^n^ analysis of *m*/*z* 727 (CL(72:4)). (**B**) Fragmentation map of CL(72:4). Fragments resulting from MS2 analysis are depicted in green. Double bond isomer fragments diagnostic of Δ9 and Δ11 unsaturation are depicted in blue and orange, respectively. (**C**) Boxplots comparing unsaturation ratios for FA(18:1) chains from CL species containing at least 3 FA(18:1) chains at *m*/*z* 713 (**upper left**), 727 (**upper right**), 738 (**bottom left**) and 739 (**bottom right**) among NL (N = 8), AST grade 1 (N = 3), AST grade 2 (N = 5), and GBM (N = 8) mitochondrial lipid extracts. Shapiro-Wilk tests were used to determine normality of distributions to inform use of non/parametric testing. *t*-tests (*m*/*z* 713, 727, 738) and Wilcoxon (*m*/*z* 739) for pairwise hypothesis tests and Holm adjustment for multiple comparisons. All pairwise comparisons were performed, only significant results are displayed (* *p* ≤ 0.05, ** *p* ≤ 0.01, *** *p* ≤ 0.001).

Due to the high degree of structural heterogeneity possible among CL molecular species and the functional implications of such heterogeneity in lipids, we characterized the structural isomer ratio composition of selected CL molecular species that contributed to separation in our PCA of NL, AST, and GBM CL profiles. Mitochondrial lipid extracts from NL, AST, and GBM samples were analyzed with ESI-CID-MS and ESI-CID-UVPD-MS [29]. Representative ESI-MS spectra of mitochondrial lipid extracts from NL, AST, and GBM tissues can be found in Figure S7. We observed a high degree of structural heterogeneity even among isobaric species, and as such we compared FA isomers among the most abundant species for two CL molecular species, and double bond isomer ratios for CL species containing at least 3 FA 18:1 chains to avoid unambiguous double bond localization [29,37,38]. Double bond isomer ratios were determined for the CL species at *m*/*z* 713.494, *m*/*z* 727.509, *m*/*z* 738.501 and *m*/*z* 739.505 comprised of three FA(18:1) chains and differing by the number of carbons and unsaturation in the fourth FA chain (Figure 3A,B). A significant increase in the ratio of Δ9 to Δ11 unsaturation was observed for all four of the CL species among AST grade 2 tissues compared to NL (all *p* < 0.05) and for three of the CL species compared to GBM tissues (all *p* < 0.01, Figure 3C). Significant differences were also observed between AST grade 1 and 2 for species at *m*/*z* 713.494, *m*/*z* 727.509, and *m*/*z* 739.505 (all *p* < 0.05). There was no significant difference in the unsaturation ratio for *m*/*z* 738.501 among AST grade 1, 2, and GBM mitochondrial extracts.

### 3.3. Immunofluorescence Microscopy Shows Differences in Mitochondrial Distribution among NL, AST, and GBM Tissues

**Figure 4:**
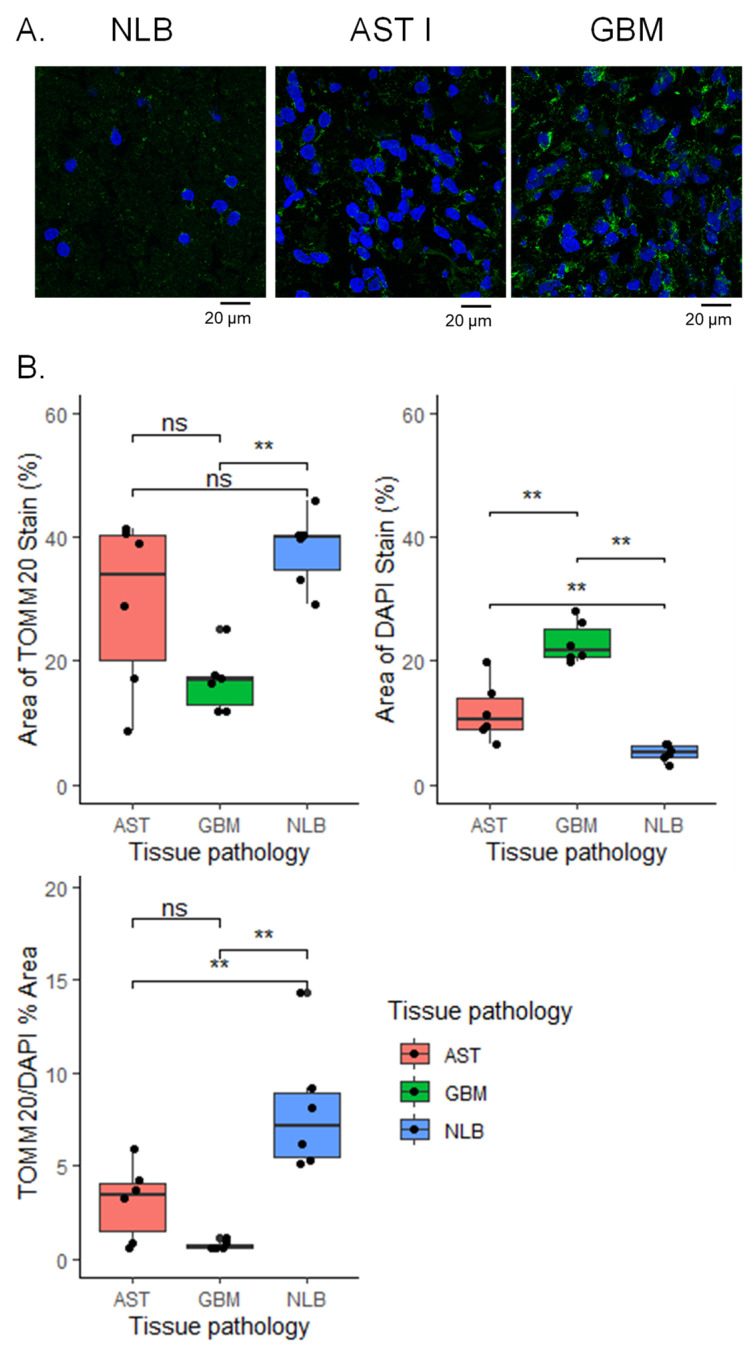
Changes in mitochondrial and cellular density are observed in immunofluorescence images of AST grade I and GBM tissues versus NLB tissue. (**A**) Representative confocal images for NL, AST grade 1, and GBM tissues stained with anti-mitochondrial antibody (green) and DAPI nuclear stain (blue). Scale bar = 20 µm. Brightness was increased uniformly by 20% for both channels in all images. (**B**) Area fraction analysis comparing area % positive for DAPI nuclear staining (**top left**), area % positive for TOMM20 staining (**top right**) and TOMM20 area % normalized by DAPI area % (**bottom left**). For NL (N = 3), AST 1 (N = 3), and GBM (N = 3), area fraction ratios obtained from 2 regions of interest were averaged from regions of pure tumor cells or normal grey matter. Wilcoxon with holm adjustment for multiple comparisons was used to determine differences among groups. ** *p* ≤ 0.01; ns, not significant.

To better understand the distribution of mitochondria in the tissues analyzed by DESI-FAIMS-MS, we performed immunofluorescence staining on a subset of tissues. Because increased cellularity is a hallmark of astrocytoma diagnosis, we were interested to observe a decrease in CL diversity and abundance among tumor specimens. As observed in the confocal microscopy images (Figure 4A), mitochondria from NL tissues were more homogenously distributed compared to tumor tissues. Accumulation of mitochondria in sparse clusters was observed in pure tumor regions from GBM tissues. AST grade 1 tissues also showed mitochondrial accumulation; however, clusters were smaller and more homogenously distributed than those observed in GBM tissues. Mitochondrial stain area % normalized to nuclear area % was compared, and significant differences were observed among AST 1 versus NL (Wilcoxon with Holm adjustment, P_adj_ = 0.017) and GBM tissues compared to NL (Wilcoxon with Holm adjustment, P_adj_ = 0.0065) (Figure 3B). The P and P_adj_ values for the comparisons performed can be found in Table S3. Intratumor mitochondrial distribution among regions of differing histology was also compared. Confocal images from patient sample 60 (discussed in previous section), which contained distinct regions of pure tumor and infiltrating tumor, are shown in Figure S8A,B. Mitochondrial aggregation was more pronounced in regions of pure tumor compared to infiltrating tumor. As expected, nuclei from the pure tumor region were more numerous and densely packed compared with those observed in the infiltrating tumor region. A significant increase in median mitochondrial stain area % per nuclear area % was observed in infiltrating tumor regions (paired *t*-test, P = 0.026) (Figure S8C). 

### 3.4. Differences in Expression of Genes Encoding Mitochondrial Enzymes Involved in Energy Production among AST 2, 3, and GBM

**Figure 5:**
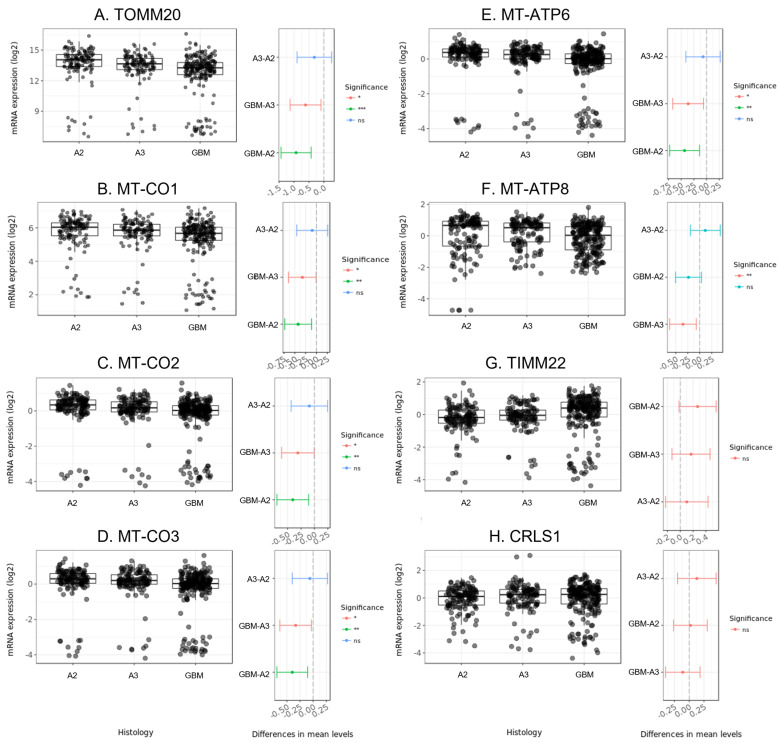
mRNA expression of mitochondria-associated protein encoding genes among primary human adult astrocytoma and glioblastoma tumors for (**A**) TOMM20, (**B**) MT-CO1, (**C**) MT-CO2, (**D**) MT-CO3, (**E**) MT-APT6, (**F**) MT-ATP8, (**G**) TIMM22, and (**H**) CRLS1. Boxplots (**right**) show transformed mRNA expression levels for astrocytomas grades 2 (N = 131), 3 (N = 120) and glioblastomas (N = 225) retrieved from the CGGA gene profile library and visualized using the GlioVis data visualization application. Differences in mean levels among pairs and the 95% confidence interval are visualized in whisker plots (**right**). Significant differences were determined using Tukey’s Honest Significant Difference. *** *p* < 0.001; ** *p* < 0.01; * *p* < 0.05; ns, not significant.

Because we observed differences in CL molecular diversity, we were interested to understand if there was evidence for alteration in enzymes involved in CL biosynthesis in mitochondrial function in astrocytoma. We considered only human primary brain tumors with AST 2 (A2, N = 131), AST 3 (A3, N = 120), and GBM (N = 225) histologies for 9 enzyme encoding-genes: TOMM20; MT-CO1, MT-CO2, and MT-CO3, encoding cytochrome c oxidase subunits; MT-ATP6, MT-ATP8, encoding ATP synthase subunits; TIMM22 encoding an inner mitochondrial membrane translocase; and CRLS1, which catalyzes CL synthesis. A table containing summary statistics of mRNA expression levels of these genes and pairwise post-hoc *t*-tests among histological groups considered can be found in Table S4. Significant decreases in mRNA expression levels were observed for GBM versus both grades of AST for TOMM20, MT-CO1-3, and MT-ATP 6 (Figure 5A–E). MT-ATP8 expression levels were found to be significantly lower in GBM versus AST 2, but no difference was observed among other pairwise comparisons (Figure 5F). No significant difference in expression levels were observed among histologies for TIMM22, and CRLS1 (Figure 5G–H).

## 4. Discussion

Complementary to the results described here using DESI-FAIMS-MS, numerous lipid classes have been reported at significantly altered abundance in glial tumors when compared to NL tissues [39,40,41], including CL in a murine xenograft model of glial cell tumors. Our findings of CL profile abnormalities in human AST and GBM tissues revealed that shorter chain, saturated FA (largely palmitic and stearic acid) containing CL, characteristic of immature CL [5,42], is observed in both NL and glioma tissues, whereas the diversity of longer chain CL species, indicative of mature CL, is decreased in tumors. These results are consistent with the trends observed in patient-derived xenograft models, where deficiencies in mature CL species, including CL(76:10) and CL(78:12), were predominantly detected in tumors versus non-tumor cortex [14]. Acyl chain heterogeneity primarily arises after biosynthesis when immature CL species undergo de-acylation and re-acylation or transacylation reactions, where cellular phospholipids donate acyl chains for incorporation in CL and is thus associated with cellular phospholipid composition [5,43]. Additional work should determine the impact of cellular phospholipid diversity on CL diversity as it relates to astrocytoma tumorigenesis and the tumor microenvironment.

It should be noted that the spatial resolution of DESI-MS imaging is not sufficient to determine if the CL profiles are uniquely characteristic of infiltrating tumor cells or adjacent cortex cells, or both. However, because diversity among longer-chain CL was observed in regions of infiltrating tumors, we suggest that at least a subset of cells in these regions contain mitochondria with more normal-like CL composition. In regions of GBM tumor tissues with decreasing tumor viability and necrosis, we observed a correlated decrease in the S/N of CL species detected, suggesting that these species are at lower abundance in necrotic regions of these tumors, though the underlying cause of this decrease was not investigated and is outside the scope of this study. The trends that we observed in CL profile diversity correlated with histology of the tumor microenvironment in AST and GBM suggest spatial differences in chemical environments in the mitochondrial membranes in these tumors.

Confocal microscopic analysis showed spatial aggregation of mitochondria in AST and GBM tissues compared with NL tissues, while a more homogenous distribution of mitochondria was observed in normal brain tissues (Figure 3A). Ultrastructural changes to mitochondrial morphology have been previously observed in AST and GBM human cells, including mitochondrial swelling and derangement in cristae structure [4,44]. Significant decreases in CL content per mitochondria have also been observed for tumor mitochondria isolated from glioma xenograft tissues [14]. As CL is known to contribute to mitochondrial membrane and cristae morphology [5], it is possible that the CL diversity we observed in these tumors is related to the cellular and tissue distribution of mitochondria as visualized in the immunofluorescence images.

Differences in FA isomer composition of CL have been observed across anatomical regions of rat brain, suggesting functional implications for this structural diversity in healthy cells [22]. Additionally, we observed changes in double bond isomer ratios among AST mitochondria, which contained CL species with a higher degree of Δ9 unsaturation versus Δ11 in comparison with NL and GBM. Significant differences in double bond isomer composition among other glycerophospholipid classes have been observed in human breast cancer tissues [45], and among anatomical regions of human cerebellar tissues and papillary thyroid carcinoma [38]. Double bond location in phosphatidylcholine has been shown to affect numerous plasma membrane properties such as area-per-lipid, membrane hydration, and membrane thickness [46]. Variations in CL double bond position in AST tissues could potentially have similar biological implications, which would affect mitochondrial membrane structure and function in these tissues. It is interesting to note that significant differences in structural composition were not observed among all CL molecular species investigated, nor were changes noted among NL and GBM tissues in any of the isobaric species we investigated. It is possible that NL and GBM tissue heterogeneity obscured alterations in structural composition. Due to the low overall abundance of CL species in DESI-FAIMS mass spectra, mapping the relative structural isomer composition with the platform utilized in this work was not possible. Nevertheless, our work allowed identification of significant differences in double bond isomer ratios in key species that we expect to provide targets for further spatially registered investigation of changes in structural CL isomer composition.

CL is known to stabilize ETC-associated protein super complexes localized to the mitochondrial membrane, as well as maintain the electrochemical gradient across the membrane, significantly improving the efficiency of oxidative phosphorylation [5]. In glioma xenograft models, decreased diversity of longer chain, mature CL was found to be directly related to altered ETC-associated protein activities in mitochondria [14]. To understand differences in mitochondria among low-grade AST and GBM tumors, we compared gene expression profiles of several genes encoding mitochondria-associated enzymes from the CGGA RNA-seq data set in the GlioVis platform (Figure 4). The observed decreases in mRNA expression levels of ETC-related enzymes MT-ATP6 and 8 and MT-CO1-3 in GBM versus lower-grade AST, while other genes encoding mitochondrial trans-membrane enzymes were not found to be differently expressed among these tumors, warrant further characterization of both gene expression and enzyme levels in these tumors to understand how decreased diversity of longer chain CL species impacts tumor growth and progression.

## 5. Conclusions

This study showcases the utility of ambient ionization mass spectrometry coupled with ion mobility for imaging of low abundance, multiply charged molecules such as CL in diverse tissue microenvironments. Our results provide spatially registered CL molecular profiles associated with mitochondrial dysfunction in histologically diverse glioma microenvironments. In particular, our results provide evidence that immature CL with shorter acyl chains are present in GBM tissues at a higher relative abundance than mature, longer acyl chain CL found in NL brain tissues, which points to aberrant mitochondria membrane composition and dysfunctionality in brain tumors. Future work will be performed to improve the analytical performance of the method to further characterize CL structural heterogeneity in a spatially resolved manner. As mitochondrial dysfunction and CL profile aberration are broadly observed in cancerous tissues, we suggest that DESI-FAIMS-MS imaging may be suitable for obtaining spatially registered CL profile information from other cancerous tissues presenting histological heterogeneity.

## Figures and Tables

**Table 1:** Subset of representative CL and MLCL species identified using high mass resolution/high mass accuracy and tandem mass spectrometry analysis.

Measured *m*/*z* ^[a]^	Tentative Attribution ^[b]^	FA Composition of Major Species	Exact *m*/*z*	Mass Error (ppm) ^[c]^	Proposed Formula
582.379	MLCL(52:2) ^2−^		582.379	0.2	C_61_H_114_O_16_P_2_
593.372	MLCL(54:4) ^2−^		593.371	1.2	C_63_H_112_O_16_P_2_
606.38	MLCL(56:5) ^2−^		606.379	1.5	C_65_H_114_O_16_P_2_
618.38	MLCL(58:8) ^2−^		618.379	1.4	C_67_H_114_O_16_P_2_
**712.487**	[CL(70:5)-2H] ^2−^	18:2_18:1_18:1_16:1	712.487	0.4	C_79_H_144_O_17_P_2_
		18:2_18:2_18:1_16:0			
		18:2_18:1_18:1_16:1			
**713.494**	[CL(70:4)-2H] ^2−^	18:2_18:1_18:1_16:0	713.495	0.7	C_79_H_146_O_17_P_2_
		18:1_18:1_18:1_16:1			
		18:2_18:1_18:1_16:0			
**723.480**	[CL(72:8)-2H] ^2−^	18:2_18:2_18:2_18:1	723.479	1.7	C_81_H_142_O_17_P_2_
		20:4_18:2_18:1_16:1			
**725.494**	[CL(72:6)-2H] ^2−^	18:2_18:2_18:1_18:1	725.495	0.7	C_81_H_146_O_17_P_2_
		18:2_18:2_18:1_18:1			
**727.509**	[CL(72:4)-2H] ^2−^	18:1_18:1_18:1_18:1	727.51	1.5	C_81_H_150_O_17_P_2_
**737.494**	[CL(74:8)-2H] ^2−^	20:4_18:2_18:1_18:1	737.495	0.7	C_83_H_146_O_17_P_2_
**738.502**	[CL(74:7)-2H] ^2−^	20:3_18:2_18:1_18:1	738.502	0.4	C_83_H_148_O_17_P_2_
		20:4_18:1_18:1_18:1			
**739.509**	[CL(74:6)-2H] ^2−^	20:4_18:1_18:1_18:1	739.51	1.5	C_83_H_150_O_17_P_2_
749.494	[CL(76:10)-2H] ^2−^		749.495	0.7	C_85_H_146_O_17_P_2_
762.501	[CL(78:13)-2H] ^2−^		762.502	1.3	C_87_H_148_O_17_P_2_

CL = cardiolipin and MLCL = monolysocardiolipin, FA = fatty acid. **^[a]^** Bolded species were identified with tandem MS experiments (CID and/or CID/CID) in addition to high mass resolution/high mass accuracy, all other species were identified by high mass resolution/high mass accuracy. **^[b]^** Lipid (X:Y) notation indicates the total number of carbons (X) and double bonds (Y) in the fatty acid chains. **^[c]^** Mass error was calculated with the exact monoisotopic *m*/*z* of the doubly deprotonated form of the assigned molecular formula.

## Data Availability

The data presented in this study are available upon request at the following repository: https://doi.org/10.7910/DVN/HY8AEX, Harvard Dataverse. Publicly available datasets analyzed in this study can be found here: [https://doi.org/10.1038/sdata.2017.24].

## Supplementary Materials

The following supporting information can be downloaded at: https://www.mdpi.com/article/10.3390/cancers15102842/s1, Figure S1: MS^2^ and MS^3^ CID mass spectra for structural identification of CL; Figure S2: Representative DESI-FAIMS spectra of MLCL detected from analysis of normal brain tissue; Figure S3: Median S/N of detected CL species from NL, AST grade I and II, and GBM regions of pure tumor or normal tissue; Figure S4: 95% confidence ellipses overlaid on scatterplot of individual PC scores; Figure S5: PCA score and PC2 loading plot for comparison of CL profiles from IDH1 mutant and wild-type specimens and boxplots comparing selected CL abundances among IDH1 mutant and wild-type specimens; Figure S6: Representative DESI-FAIMS mass spectra of CL detected from different histological regions of GBM tissues; Figure S7: Representative ESI-MS spectra of mitochondrial lipid extracts from NL, AST, and GBM tissues; Figure S8: Immunofluorescence confocal images and area % analysis of distinct histological regions from patient tumor 60; Table S1: List of patient samples and demographics; Table S2: Table of complete list of CL species detected by DESI-FAIMS-MS analysis; Table S3: Post hoc Wilcoxon with Holm adjustment results for immunofluorescent stain area fraction analysis; Table S4: *p*-values for pairwise comparisons of gene expression levels.
