# Supplementary material for: Mass Spectrometry Imaging Reveals Abnormalities in Cardiolipin Composition and Distribution in Astrocytoma Tumor Tissues
Source: Cancers (Basel). 2023 May 19;15(10):2842. doi: 10.3390/cancers15102842 (PMC10216144; doi:10.3390/cancers15102842)
Supplement: Supplementary file 1 [file cancers-15-02842-s001.zip › cancers-2297472-supplementary.pdf]

SUPPLEMENTAL FIGURES AND TABLES

Mass Spectrometry Imaging reveals abnormalities in Cardiolipin composition and distribution in Astrocytoma Tumor Tissues

Anna C. Krieger[a], Luis A. Macias[a], J. Clay Goodman[b], Livia S. Eberlin[a]*

[a] Department of Chemistry, The University of Texas at Austin, Austin, TX.

[b] Departments of Pathology & Immunology and Neurology, Baylor College of Medicine, Houston, Texas, USA.

* *to whom correspondence should be addressed*: liviase@utexas.edu

Contents:

Supplemental Materials and Methods.

Supplemental Results.

Table S1. List of patient samples and demographics.

Figure S1. MS^2 and MS^3 CID mass spectra for structural identification of CL

Figure S2. Representative DESI-FAIMS spectra of MLCL detected from analysis of normal brain tissue.

Table S2. Table of complete list of CL species detected by DESI-FAIMS-MS analysis

Figure S3. Median S/N of detected CL species from NL, AST grade I and II, and GBM regions of pure tumor or normal tissue.

Figure S4. 95% confidence ellipses overlaid on scatterplot of individual PC scores.

Figure S5. PCA score and PC2 loading plot for comparison of CL profiles from IDH1 mutant and wild-type specimens and boxplots comparing selected CL abundances among IDH1 mutant and wild-type specimens.

Figure S6. Representative DESI-FAIMS mass spectra of CL detected from different histological regions of GBM tissues.

Table S3. Post hoc Wilcoxon with Holm adjustment results for immunofluorescent stain area fraction analysis.

Figure S7. Representative ESI-MS spectra of mitochondrial lipid extracts from NL, AST, and GBM tissues.

Figure S8. Immunofluorescence confocal images and area % analysis of distinct histological regions from patient tumor 60.

Table S4. P-values for pairwise comparisons of gene expression levels.

Supplemental Material and Methods

FAIMS. An ultraFAIMS device (Owlstone Ltd., Cambridge, UK) using an ND chip (Owlstone), was used for all experiments. Dispersion field (DF) and compensation field (CF) values were optimized for doubly deprotonated CL S/N using mouse brain tissues, and were determined to be 220 and 2.25 Td, respectively. During DESI-FAIMS-MS image acquisition, the ultraFAIMS was operated in static mode at optimized CF and DF values. To facilitate data comparison experiments, DESI source optimization was maintained for sequential DESI-FAIMS-MS and DESI-MS analysis of serial sections.

CID-UVPD-MS. CID-UVPD experiments were performed to evaluate differences in unsaturation location within CL acyl chains. Due to significant structural heterogeneity among CL isobars and isomers, unambiguous localization of double bonds on all acyl chains was unattainable. Instead, double bond isomer ratios were determined for the CL species at *m/z* 713.494, *m/z* 727.509, *m/z* 738.501 and *m/z* 739.505 comprised of three FA(18:1) chains and differing by the number of carbons and unsaturation in the fourth FA chain. As shown in **Figure S5A**, the first fragmentation event results in the loss of the fourth, dissimilar FA chain. This fragment ion is then further fragmented by UVPD to determine the relative prevalence of double bonds at the Δ9 and Δ11 position among the remaining three FA(18:1) chains.[29] The intensities of diagnostic fragment ions associated with each double bond position are summed and the ratio (Rel. $C_{\Delta 9}/C_{\Delta 11} = (I_{1035} + I_{1059})/(I_{1063} + I_{1087})$) was then compared among NL, AST, and GBM tissues.

Supplemental Results

Coupling FAIMS with DESI-MS improves sensitivity for CL. Negative ion mode DESI-MS and DESI-FAIMS-MS were used to analyze serial sections of 17 human normal brain tissue samples. Both DESI-MS and DESI-FAIMS-MS analysis of normal brain tissue sections produced mass spectra (**Fig. S9A**) containing a variety of lipid species including the highly abundant ceramide (36:1) (*m/z* 600.511), phosphatidylethanolamine (38:3) (*m/z* 790.536), phosphatidylserine (38:3)

(*m/z* 834.525), and phosphatidylinositol (38:4) (*m/z* 885.546), previously observed in human brain tissues by DESI-MS.[34, 35] DESI-FAIMS mass spectra were also characterized by these diverse lipid species at an overall lower absolute abundance, however a distinct profile of ions spaced by 0.5 *m/z* difference in the mass range *m/z* 690-780 putatively attributed to CL species was observed at much higher relative abundance compared with DESI mass spectra.

The median number of CL molecular species detected in DESI-FAIMS-MS analysis of NL brain tissues was 30, a significant increase compared to a median of 21 species detected by DESI-MS alone (P = 0.00032, paired Wilcoxon) (**Fig. S9B**). Most notably, though the average total abundance detected per-pixel for CL (74:8) (*m/z* 737.495), a major species in normal human brain tissue, decreased by 47%, the average S/N ratio increased 13-fold when comparing DESI-FAIMS-MS to DESI-MS analysis. In a single MS scan, DESI-FAIMS-MS allowed detection of up to 32 *m/z* values corresponding to CL species, and the detection of 46 unique molecular species among the analyzed samples. Thus, DESI-FAIMS-MS was used for all imaging analyses to maximize sensitivity and diversity of CL molecular species detected.

MS^n analysis to determine differences in CL molecular structure among NL, AST, and GBM tissues. To elucidate differences in structural composition of isobaric CL species among NL, AST, and GBM tissues, MS^n experiments were performed on the mitochondrial lipid extracts using a tandem CID and UVPD MS technique. ESI mass spectra of mitochondrial lipid extracts show CL profile alterations similar to DESI-FAIMS mass spectra from NL, AST, and GBM tissues (**Fig. S10**), confirming that alterations in CL composition occurs in the mitochondria from these tissues.

Interestingly, another series of doubly charged ions were observed at lower relative abundance in the DESI-FAIMS mass spectra from *m/z* 580-630 (**Fig. S2**). These ions were tentatively assigned as monolysocardiolipin (MLCL) species, although their low abundance

precluded imaging and structural confirmation by MS^n analysis. Note that the MLCL species detected at low relative abundance in NL samples were not detected in DESI-FAIMS mass spectra of AST or GBM tissues. A full list of detected CL and species tentatively assigned as MLCL can be found in **Table S2**. MLCL molecular species were detected in ESI mass spectra from NL, AST, and GBM tissues.

Differences in acyl chain composition were determined for NL (N = 4), AST grade 2 (N = 4), and GBM (N = 4) tissues. Two CL species detected in DESI-FAIMS mass spectra of all tissue types, a shorter chain species *m/z* 713.494 and a longer chain species *m/z* 738.501, were subjected to tandem MS analyses. Representative fragmentation spectra and statistical analyses can be found in **Figure S8**. For *m/z* 713.494, two major CL species were identified, CL(18:1_18:1_18:1_16:1) with PA-loss fragments at *m/z* 727.49 (M-PA(18:1_18:1)$^{-1}$) and *m/z* 755.52 (M-PA(18:1_16:1)$^{-1}$), and CL(18:2_18:1_18:1_16:0) with fragments at *m/z* 729.51 (M-PA(18:2_18:1)$^{-1}$) and *m/z* 753.51 (M-PA(18:1_16:0)$^{-1}$). Comparing the ratio of average fragment signal intensity for CL(18:1_18:1_18:1_16:1) versus CL(18:2_18:1_18:1_16:0), we observed a borderline significant difference between NL and AST tissues, where AST tissues had a greater abundance of CL(18:1_18:1_18:1_16:1) relative to CL(18:2_18:1_18:1_16:0) compared to NL tissues (Kruskal-Wallis, p = 0.057). No significant difference was observed for other pairwise comparisons. For the longer chain CL species *m/z* 738.5015, two major CL isomers were identified, CL(20:3_18:1_18:2_18:1) and CL(20:4_18:1_18:1_18:1). The ratio of average abundances of the fragment ions resulting from PA-losses were again compared, and AST tissues had a greater abundance of CL(20:3_18:1_18:2_18:1) relative to CL(20:4_18:1_18:1_18:1) compared to NL (Kruskal-Wallis, p = 0.029) and GBM tissues (Kruskal-Wallis, p = 0.029).

The mean area % of positive mitochondrial and nuclear stain was compared among NL, AST 1, and GBM tissues (**Fig. S4B**). A significant increase in mitochondrial stain area % was observed in NL and AST 1 samples compared with GBM (P_{adj} = 0.065, Wilcoxon with Holm

adjustment for multiple comparisons). A significant increase in nuclear stain area % was observed in AST 1 1versus NL (P_{adj} = 0.0065) and GBM versus NL tissues (P_{adj} = 0.0065), and GBM tissues had greater nuclear area % versus AST 1 (P_{adj} = 0.0043).

Supplementary Table S1. List of patient samples and demographics.[a]

Patient #	Age	Race	Sex	Site	Diagnosis[b]	IDH1 status[c]	WHO grade[b]
1	78	B	F	Brain, unspecified loc			N/A
2	78	B	F	Brain, unspecified loc			N/A
3	72	W	M	Brain, unspecified loc			N/A
4	20	H	M	Brain, unspecified loc			N/A
5	78	W	F	Brain, unspecified loc			N/A
6	59	W	M	Brain, unspecified loc			N/A
7	46	W	M	Brain, unspecified loc			N/A
8	49	W	F	Brain, unspecified loc			N/A
9	39	B	M	Brain, unspecified loc			N/A
10	71	W	M	Brain, unspecified loc			N/A
11	82	W	F	Brain, unspecified loc			N/A
12	58	W	F	Brain, unspecified loc			N/A
13	62	W	M	Brain, unspecified loc			N/A
14	49	B	M	Brain, unspecified loc			N/A
15	63	W	M	Brain, unspecified loc			N/A
16	68	W	M	Brain, unspecified loc			N/A
17	25	W	M	Brain, unspecified loc			N/A
18	54		F	Brain, unspecified loc			N/A
19	54		F	Brain, temporal gyrus			N/A
20	37		M	Brain, unspecified loc			N/A
21	23		M	Brain, unspecified loc			N/A
22	34		M	Brain, unspecified loc			N/A
23	27		M	Brain, unspecified loc			N/A
24	41		F	Brain, unspecified loc			N/A
25	65		M	Brain, unspecified loc			N/A
26	19		M	Brain, unspecified loc			N/A
27	55		F	Brain, unspecified loc			N/A
28	45		M	Brain, unspecified loc			N/A
29	34		M	Brain, unspecified loc			N/A

30					Astrocytoma	Mut	II
31					Astrocytoma	Mut	II
32	30	O	F	Spinal cord	Astrocytoma, pilocytic		I
33	29		F	Brain, L frontal	Glioma, mixed		II
34	9	W	F		Astrocytoma, pilocytic		I
35	28	O	F	Brain, cerebellum	Astrocytoma, pilocytic		I
36	14	W	F		Astrocytoma, pilocytic		I
37	13	B	F		Astrocytoma, pilocytic		I
38	24	W	M		Astrocytoma, pilocytic		I
39	20	W	F	Brain, frontal	Astrocytoma, diffuse		II
40	22	B	M		Astrocytoma, pilocytic		I
41					Astrocytoma, pilocytic		I
42	18	B	F		Astrocytoma, pilocytic		I
43	12	A	M		Astrocytoma, pilocytic		I
44	26	W	M		Astrocytoma, pilocytic		I
45	54	B	M		Astrocytoma, pilocytic		I
46	39	O	M		Astrocytoma, diffuse		II
47					Astrocytoma		II
48	65	W	M		Glioblastoma		IV
49	76	W	M		Glioblastoma		IV
50	86	W	F		Glioblastoma		IV
51	37	B	F		Glioblastoma		IV
52	54	W	M		Glioblastoma		IV
53	60	W	F		Glioblastoma multiforme		IV
54	48	W	F		Glioblastoma multiforme		IV
55	69	W	M		Glioblastoma multiforme		IV
56	40	W	F		Glioblastoma multiforme		IV
57	65	W	F		Glioblastoma multiforme		IV
58	52	W	F		Glioblastoma multiforme		IV
59	17	W	F		Glioblastoma multiforme		IV
60					Glioblastoma	WT	IV
61					Glioblastoma	WT	IV
62					Glioblastoma		IV
63					Glioblastoma	WT	IV
64					Glioblastoma		IV
65					Glioblastoma	WT	IV
66					Glioblastoma		IV
67	68	W	M	Brain, temporal	Glioblastoma	WT	IV
68	81	W	F	Brain, R parietal-occipital	Glioblastoma	WT	IV
69	62	W	M	Brain, temporal	Glioblastoma, residual/recurrent	WT	IV
70	44	W	M	Brain, occipital	Glioblastoma	Mut	IV
71	59	W	M	Brain, frontal	Glioblastoma		IV
72	28	W	F	Brain, frontal	Glioblastoma, residual/recurrent	WT	IV

73	62	W	F	Brain, frontal	Glioblastoma	WT	IV
74	59	W	M	Brain, frontal	Glioblastoma	WT	IV
75	49		F	Brain, frontal	Glioblastoma, residual/recurrent	Mut	IV
76	51	W	M	Brain, frontal	Glioblastoma	Mut	IV
77	50		F	Brain, temporal	Glioblastoma	WT	IV

B = Black, H = Hispanic, W = White, O = Other, M = Male, F = Female, L = Left, R = Right, Mut = mutant, WT = wild-type, WHO = World Health Organization

[a] Missing demographic information was not provided by the sample source.

[b] Samples 1-29 are referred to as “normal” or “NL” and underwent histopathological evaluation by JCG. None of these samples contained evidence of abnormal or cancerous cells. All samples were obtained at autopsy and all patients had a history of seizures or seizure disorders.

[c] IDH1 status was determined by immunohistochemistry and was provided by sample source.

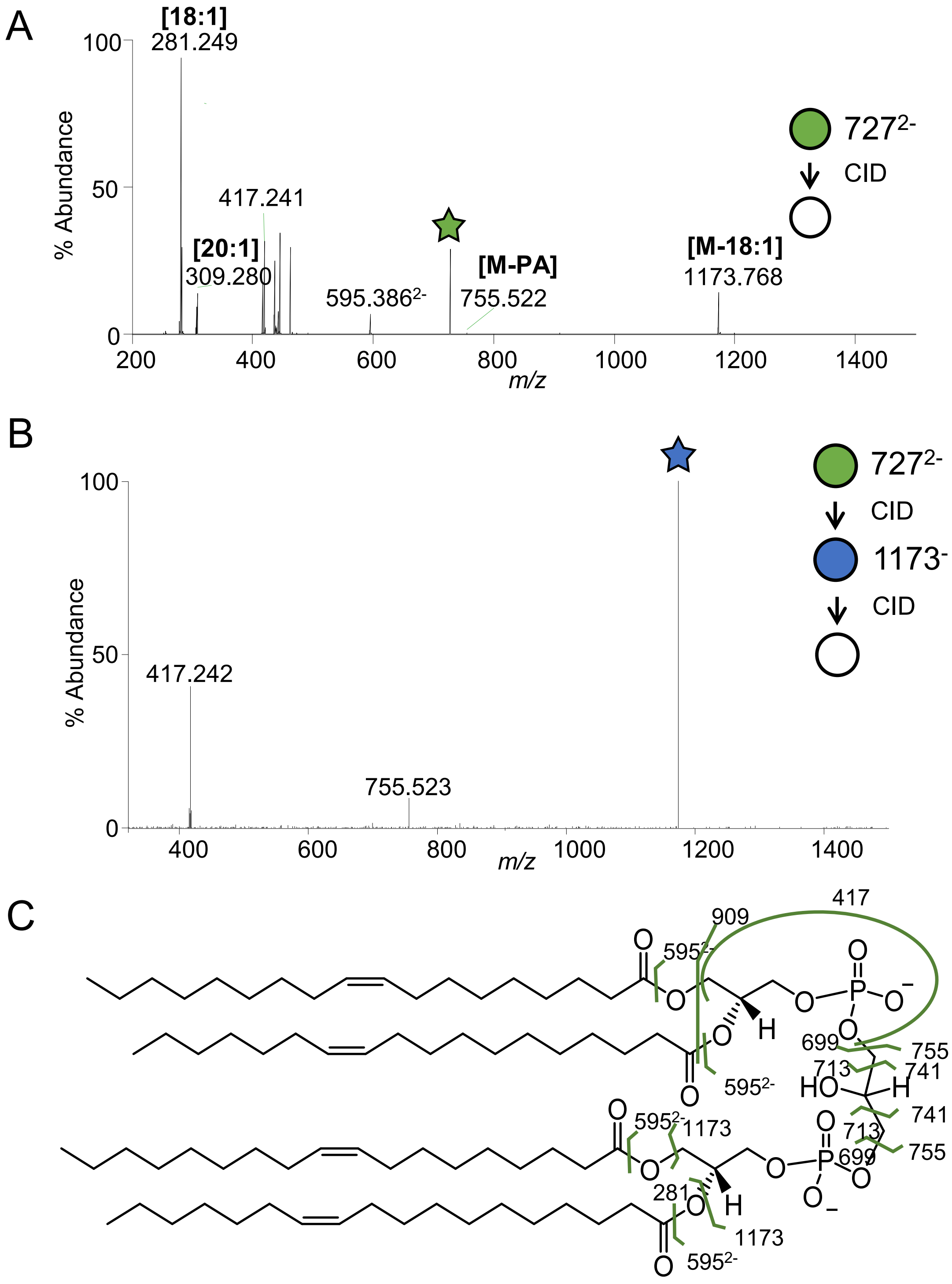

Supplementary Figure S1. Representative A) CID and B) CID-CID mass spectra for structural identification of CL detected by DESI-FAIMS-MS from human tissue sections. C) Fragmentation map associated with the above spectra.

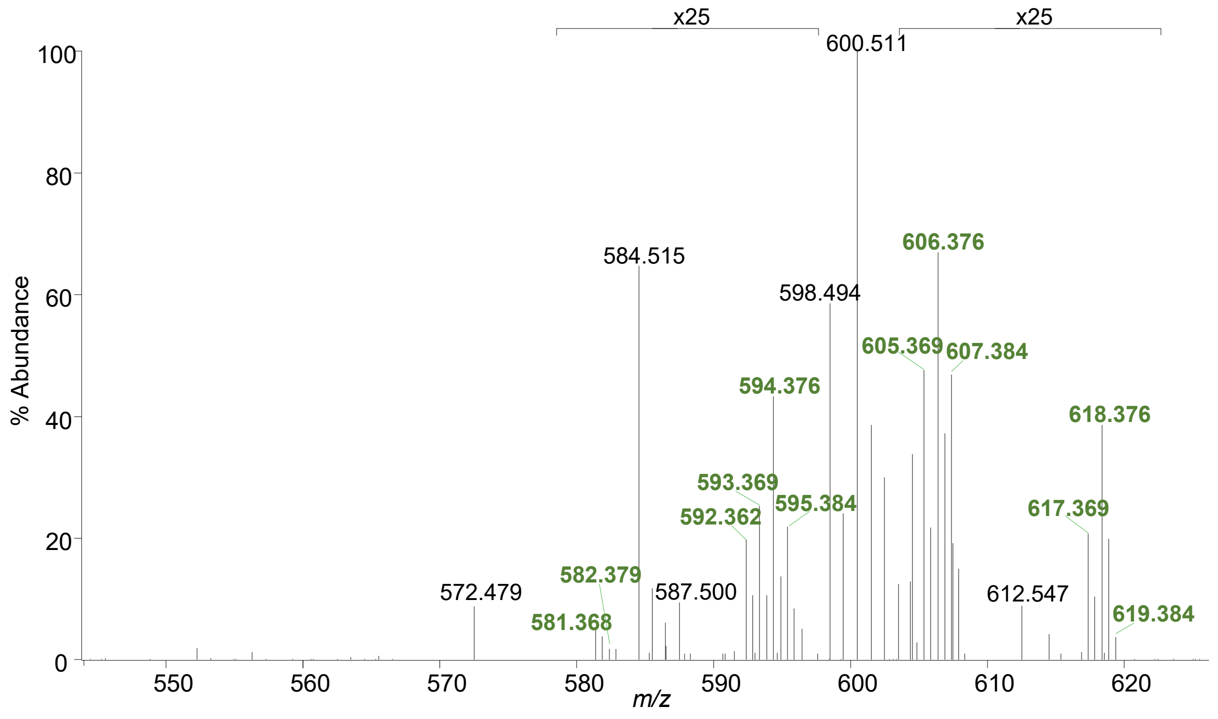

Supplementary Figure S2. Representative negative mode DESI-FAIMS mass spectra of NL tissue from *m/z* 555 – 625. Detected MLCL species are denoted by bolded green flags.

Supplementary Table S2. Full list of MLCL and CL species identified using high mass resolution/high mass accuracy and tandem mass spectrometry (CID) analysis.

Measured *m/z*[a]	Tentative attribution[b]	FA composition of major species	Exact *m/z*	Mass Error (ppm) [c]	Proposed formula
579.356*	MLCL(52:5)2-		579.356	0.6	C61H108O16P2
580.364*	MLCL(52:4)2-		580.363	0.9	C61H110O16P2
581.372	MLCL(52:3)2-		581.371	1.2	C61H112O16P2
582.379	MLCL(52:2)2-		582.379	0.2	C61H114O16P2
592.365	MLCL(54:5)2-		592.363	2.6	C63H110O16P2
593.372	MLCL(54:4)2-		593.371	1.2	C63H112O16P2
594.380	MLCL(54:3)2-		594.379	1.5	C63H114O16P2
595.387	MLCL(54:2)2-		595.387	0.1	C63H116O16P2
604.365*	MLCL(56:7)2-		604.363	2.6	C65H110O16P2
605.372	MLCL(56:6)2-		605.371	1.2	C65H112O16P2
606.380	MLCL(56:5)2-		606.379	1.5	C65H114O16P2
607.387	MLCL(56:4)2-		607.387	0.1	C65H116O16P2
608.396*	MLCL(56:3)2-		608.395	2.0	C65H118O16P2
616.363*	MLCL(58:10)2-		616.363	0.7	C67H110O16P2
617.373	MLCL(58:9)2-		617.371	2.8	C67H112O16P2
618.380	MLCL(58:8)2-		618.379	1.4	C67H114O16P2
619.389	MLCL(58:7)2-		619.387	3.3	C67H116O16P2
629.372*	MLCL(60:11)2-		629.371	1.1	C69H112O16P2
630.379*	MLCL(60:10)2-		630.379	0.2	C69H114O16P2
697.461	[CL(68:6)-2H]2-		697.463	3.2	C77H138O17P2
698.472	[CL(68:5)-2H]2-		698.471	1.4	C77H140O17P2
699.479	[CL(68:4)-2H]2-		699.479	0.3	C77H142O17P2
700.490	[CL(68:3)-2H]2-		700.487	4.7	C77H144O17P2
701.490	[CL(68:2)-2H]2-		701.495	6.4	C77H146O17P2
709.460	[CL(68:6)-2H]2-		709.463	4.5	C79H138O17P2
710.473	[CL(70:8)-2H]2-		710.471	2.8	C79H140O17P2
**711.478**	[CL(70:6)-2H]2-	18:2_18:2_18:1_16:1	711.479	1.1	C79H142O17P2
		18:2_18:2_18:1_16:1			
		18:2_18:2_18:1_16:1			
		20:4_18:1_16:1_16:0			
**712.487**	[CL(70:5)-2H]2-	18:2_18:1_18:1_16:1	712.487	0.4	C79H144O17P2

		18:2_18:2_18:1_16:0			
		18:2_18:1_18:1_16:1			
**713.494**	[CL(70:4)-2H]2-	18:2_18:1_18:1_16:0	713.495	0.7	C79H146O17P2
		18:1_18:1_18:1_16:1			
		18:2_18:1_18:1_16:0			
**714.501**	[CL(70:3)-2H]2-		714.502	1.8	C79H148O17P2
**721.461**	[CL(72:10)-2H]2-		721.463	3.0	C81H138O17P2
**722.471**	[CL(72:9)-2H]2-		722.471	0.0	C81H140O17P2
**723.480**	[CL(72:8)-2H]2-	18:2_18:2_18:2_18:1	723.479	1.7	C81H142O17P2
		20:4_18:2_18:1_16:1			
**724.487**	[CL(72:7)-2H]2-	20:4_18:1_18:1_16:1	724.487	0.4	C81H144O17P2
		20:4_18:2_18:1_16:0			
**725.494**	[CL(72:6)-2H]2-	18:2_18:2_18:1_18:1	725.495	0.7	C81H146O17P2
		18:2_18:2_18:1_18:1			
726.503	[CL(72:5)-2H]2-		726.502	1.0	C81H148O17P2
**727.509**	[CL(72:4)-2H]2-	18:1_18:1_18:1_18:1	727.51	1.5	C81H150O17P2
**728.516**	[CL(72:3)-2H]2-	18:1_18:1_18:1_18:0	728.518	2.8	C81H152O17P2
733.459	[CL(74:12)-2H]2-		733.463	5.7	C83H138O17P2
734.470	[CL(74:11)-2H]2-		734.471	1.4	C83H140O17P2
735.479	[CL(74:10)-2H]2-		735.479	0.3	C83H142O17P2
**736.486**	[CL(74:9)-2H]2-	20:4/18:2/18:2/18:1	736.487	1.0	C83H144O17P2
		20:4/20:3/18:1/16:1			
**737.494**	[CL(74:8)-2H]2-	20:4/18:2/18:1/18:1	737.495	0.7	C83H146O17P2
**738.502**	[CL(74:7)-2H]2-	20:3/18:2/18:1/18:1	738.502	0.4	C83H148O17P2
		20:4/18:1/18:1/18:1			
**739.509**	[CL(74:6)-2H]2-	20:4/18:1/18:1/18:1	739.51	1.5	C83H150O17P2
740.516	[CL(74:5)-2H]2-		740.518	2.7	C83H152O17P2
747.476	[CL(76:12)-2H]2-		747.477	1.9	C85H142O17P2
748.487	[CL(76:11)-2H]2-		748.487	0.4	C85H144O17P2
**749.494**	[CL(76:10)-2H]2-		749.495	0.7	C85H146O17P2
**750.502**	[CL(76:9)-2H]2-		750.502	0.4	C85H148O17P2
**751.509**	[CL(76:8)-2H]2-		751.51	1.5	C85H150O17P2
752.515	[CL(76:7)-2H]2-		752.518	4.0	C85H152O17P2
753.522	[CL(76:6)-2H]2-		753.526	5.0	C85H154O17P2
759.475	[CL(78:14)-2H]2-		759.479	5.0	C87H142O17P2

760.483	[CL(78:13)-2H]2-		760.487	4.9	C87H144O17P2
**761.493**	[CL(78:12)-2H]2-		761.495	2.0	C87H146O17P2
**762.500**	[CL(78:13)-2H]2-		762.502	3.0	C87H148O17P2
763.511	[CL(78:10)-2H]2-		763.51	1.2	C87H150O17P2
764.516	[CL(78:9)-2H]2-		764.518	2.6	C87H152O17P2
771.476	[CL(80:14)-2H]2-		771.479	3.6	C89H142O17P2
772.483	[CL(80:13)-2H]2-		772.487	4.8	C89H144O17P2
773.491	[CL(80:12)-2H]2-		773.495	4.5	C89H146O17P2
774.503	[CL(80:11)-2H]2-		774.502	0.9	C89H148O17P2
775.510	[CL(80:10)-2H]2-		775.51	0.1	C89H150O17P2
776.520	[CL(80:9)-2H]2-		776.518	2.6	C89H152O17P2

CL = cardiolipin, MLCL = monolysocardiolipin, FA = Fatty Acid

[a] Bolded species identified with tandem MS experiments in addition to high mass resolution/high mass accuracy measurements, all other species were identified by high mass resolution/high mass accuracy measurements. Species indicated with an asterisk (*) were detected by ESI-MS analysis only. All other species were detected in both DESI-FAIMS-MS and ESI-MS analysis.

[b] Lipid (X:Y) notation indicates the total number of carbons (X) and double bonds (Y) in the fatty acid chains.

[c] Mass error was calculated with the exact monoisotopic *m/z* of the doubly deprotonated form of the assigned molecular formula

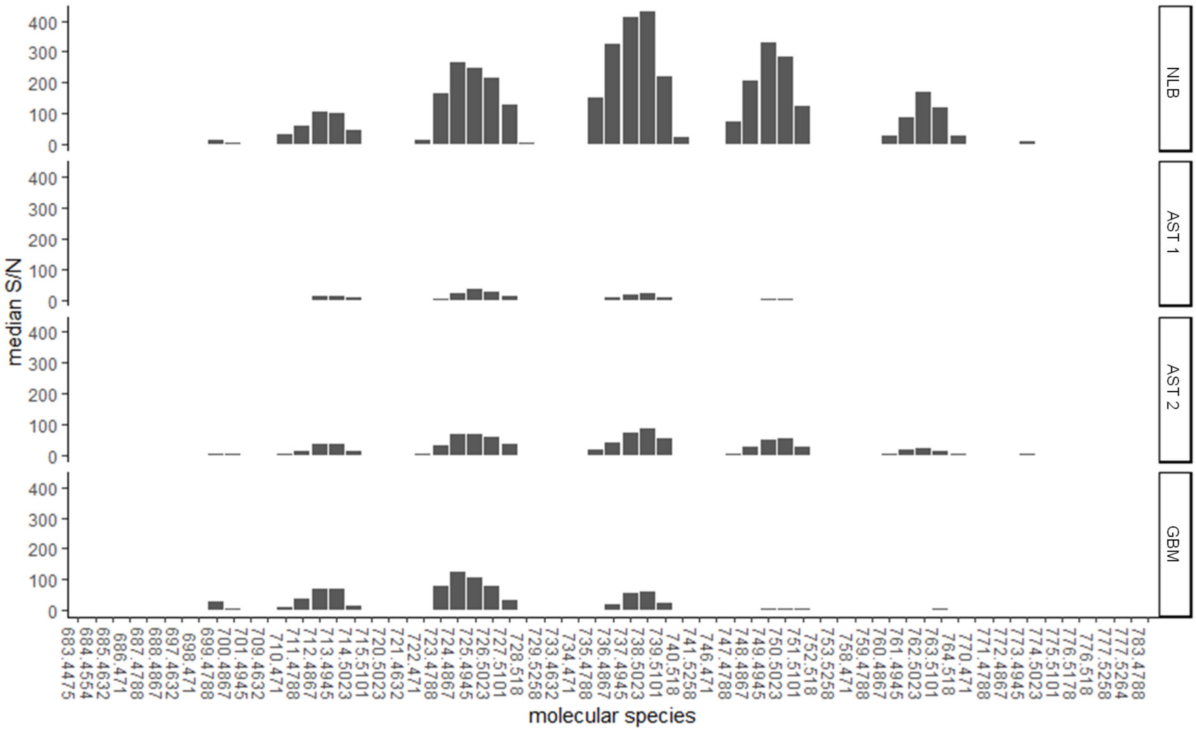

Supplementary Figure S3. Median S/N of detected CL species from NL, AST grade I and II, and GBM extracted regions (pure tumor or normal cells).

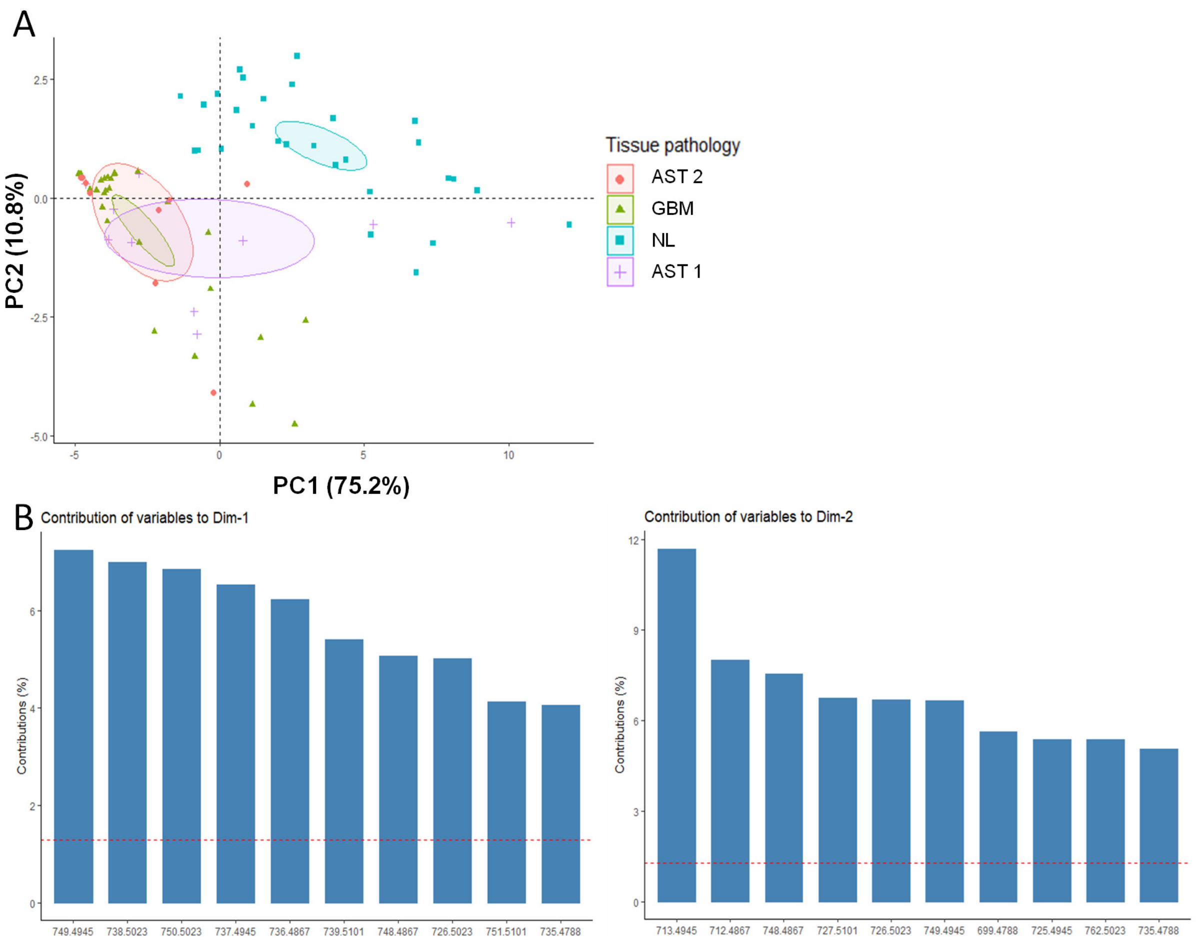

Supplementary Figure S4. A) 95% confidence ellipses overlaid on scatterplot of individual PC scores B) Variables with the highest contributions to PC1 (left) and PC2 (right). Red dotted line is expected contribution of a single feature to the PC given the number of original features.

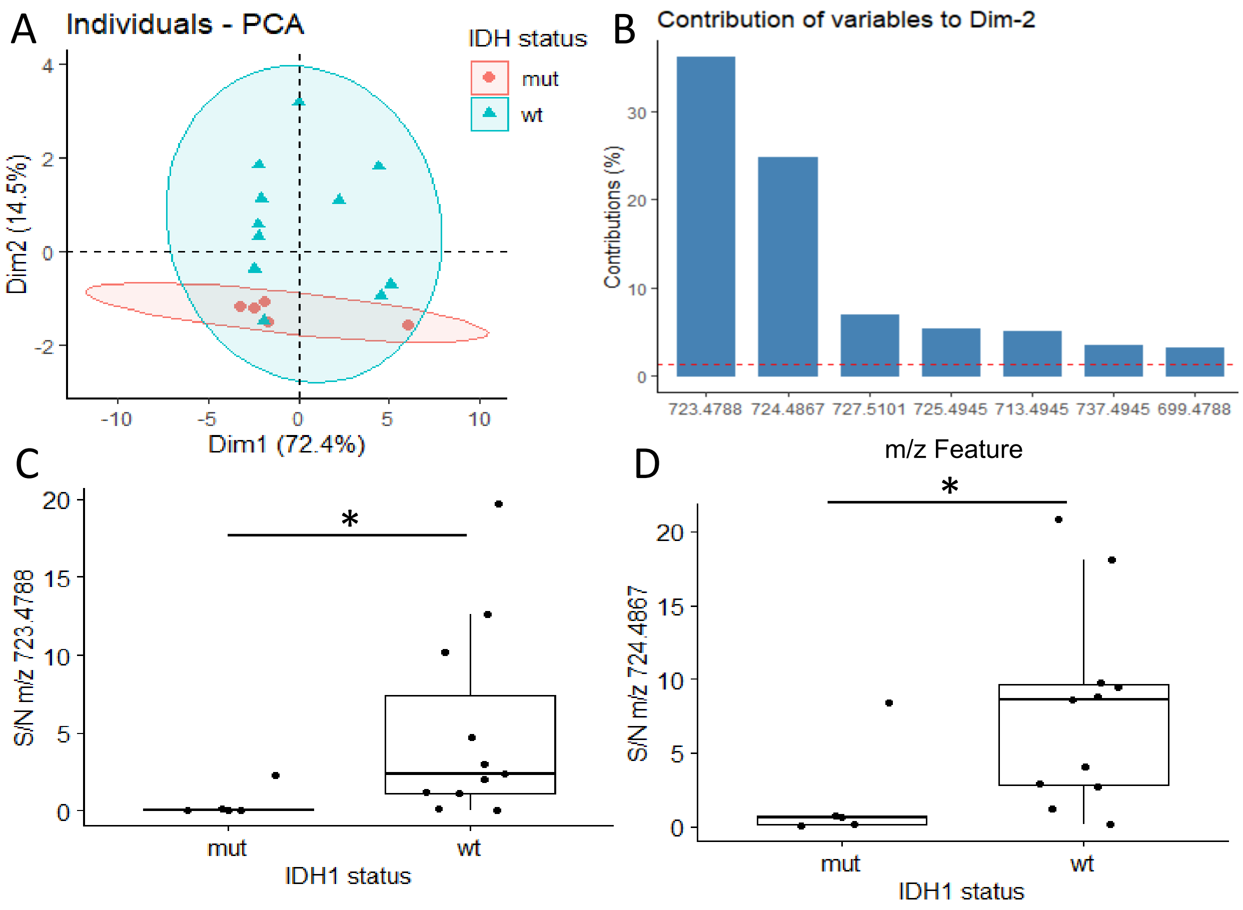

Supplementary Figure S5. PCA score plot (A) and loading plot for PC2 (B) for investigation of CL profile differences among IDH1 mutant (N = 5) and wild type (N = 11) tumors. Comparison of S/N of m/z 723.479 (C) and m/z 724.469 among IDH1 mutant and wild type tumors, (Wilcoxon with Holm adjustment, both P = 0.0275).

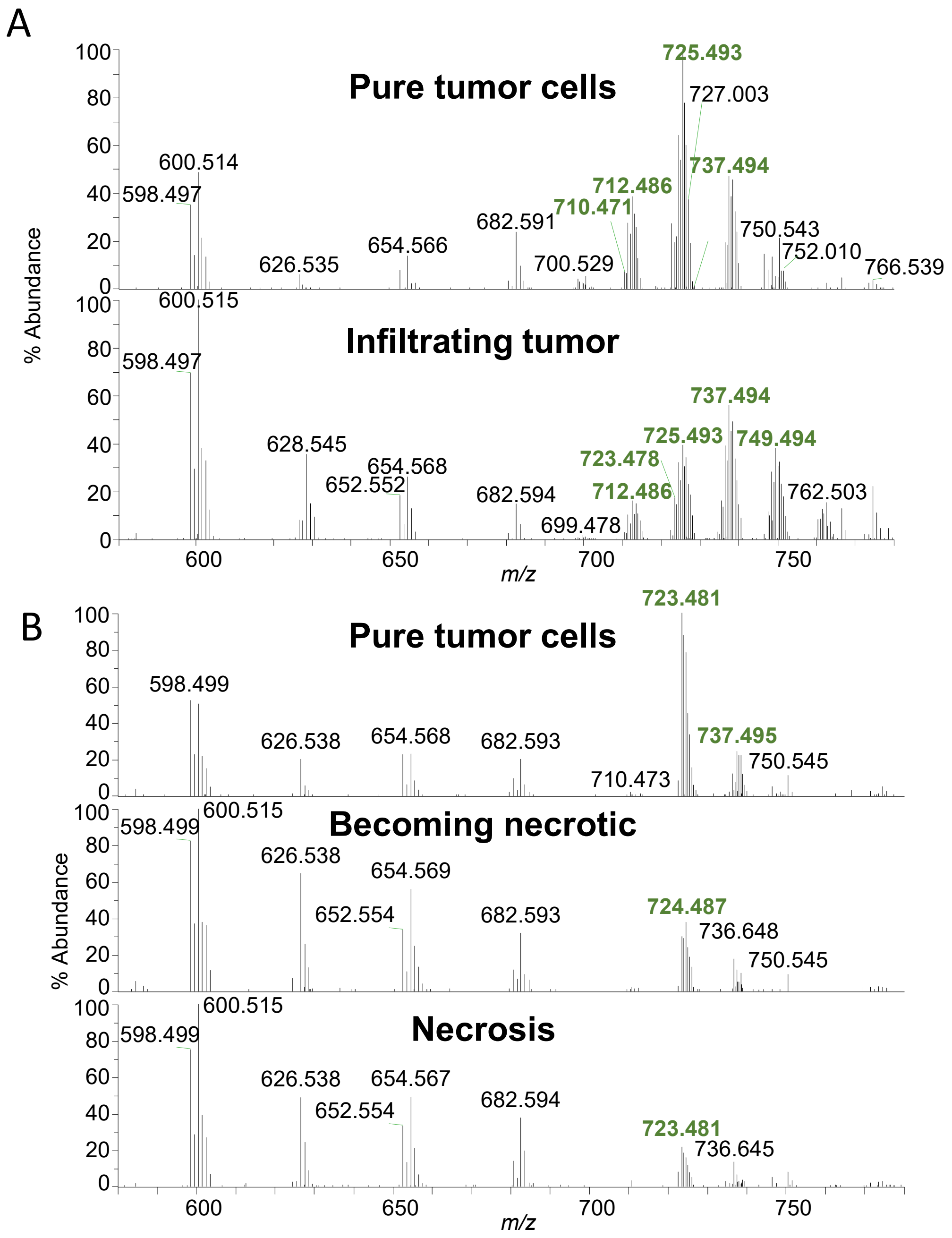

Supplementary Figure S6. Representative negative mode DESI-FAIMS mass spectra of different histological regions from *m/z* 680 – 780 for A) pure tumor (top) and infiltrating tumor (bottom) from patient sample 71. Spectra are an average of 20 scans from the same region, and B) pure tumor (top), tumor in the process of becoming necrotic (middle), and necrosis (bottom) from patient sample 60. spectra are an average of 6 scans from the same histological region. CL species are denoted by bolded green flags.

Table S3. Post hoc Wilcoxon with Holm adjustment results for immunofluorescent stain area fraction analysis.

Factor	Pairwise Comparison Group 1	Group 2	P	P_{adj}[a]	Significance Code
TOMM20 stain area %	AST 1	GBM	0.132	0.26	ns
	AST 1	NL	0.394	0.39	ns
	GBM	NL	0.00216	0.0022	**
DAPI stain area %	AST 1	GBM	0.00433	0.0065	**
	AST 1	NL	0.00216	0.0065	**
	GBM	NL	0.00216	0.0065	**
TOMM20 / DAPI stain area %	AST 1	GBM	0.0649	0.065	ns
	AST 1	NL	0.00866	0.017	**
	GBM	NL	0.00216	0.0065	**

AST 1 = astrocytoma grade 1, GBM = glioblastoma, NL = normal cortex, ns = no significance

[a] Holm adjusted for multiple comparisons

Table S4. mRNA expression level summary statistics and pairwise comparison results among tumor histologies from the CGGA dataset accessed via GlioVis.

			mRNA expression (log_2)			Pairwise t-tests		
Gene	Histology	Sample count (N)	median	mean	sd	Pairwise comparison	P (adjusted, Bonferroni)	significance
**TOMM20**	Astrocytoma (A2)	131	6.03	5.74	1.1	A2 - A3	1.00E+00	ns
	Anaplastic Astrocytoma (A3)	120	5.86	5.64	1.08	GBM - A3	5.10E-02	.
	Glioblastoma	225	5.67	5.31	1.32	GBM - A2	4.50E-03	**
**MT-CO1**	Astrocytoma (A2)	131	14.06	13.64	1.83	A2 - A3	5.90E-01	ns
	Anaplastic Astrocytoma (A3)	120	13.65	13.32	1.72	GBM - A3	1.70E-02	*
	Glioblastoma	225	13.26	12.71	2.12	GBM - A2	5.20E-05	****
**MT-CO2**	Astrocytoma (A2)	131	0.34	0.11	1.1	A2 - A3	1.00E+00	ns
	Anaplastic Astrocytoma (A3)	120	0.18	0.01	1.03	GBM - A3	5.20E-02	.
	Glioblastoma	225	0.03	-0.3	1.22	GBM - A2	4.20E-03	**
**MT-CO3**	Astrocytoma (A2)	131	0.3	0.09	1.06	A2 - A3	1.00E+00	ns
	Anaplastic Astrocytoma (A3)	120	0.2	0.03	1.01	GBM - A3	2.80E-02	*
	Glioblastoma	225	0.03	-0.31	1.23	GBM - A2	4.60E-03	**

**MT-ATP6**	Astrocytoma (A2)	131	0.38	0.11	1.1	A2 - A3	1.00E+00	ns
	Anaplastic Astrocytoma (A3)	120	0.26	0.04	1.04	GBM - A3	1.50E-02	*
	Glioblastoma	225	0.02	-0.32	1.22	GBM - A2	1.80E-03	**
**MT-ATP8**	Astrocytoma (A2)	131	0.66	0.05	1.32	A2 - A3	1.00E+00	ns
	Anaplastic Astrocytoma (A3)	120	0.52	0.17	0.89	GBM - A3	9.60E-03	**
	Glioblastoma	225	0.04	-0.19	0.94	GBM - A2	1.30E-01	ns
**TIMM22**	Astrocytoma (A2)	131	-0.18	-0.27	0.92	A2 - A3	1.00E+00	ns
	Anaplastic Astrocytoma (A3)	120	-0.04	-0.16	0.94	GBM - A3	5.50E-01	ns
	Glioblastoma	225	0.4	0	1.29	GBM - A2	8.50E-02	.
**TIMM23**	Astrocytoma (A2)	131	-0.04	-0.16	0.9	A2 - A3	1.00E+00	ns
	Anaplastic Astrocytoma (A3)	120	0.16	-0.07	1.01	GBM - A3	2.40E-01	ns
	Glioblastoma	225	0.06	-0.29	1.28	GBM - A2	8.90E-01	ns
**CRLS1**	Astrocytoma (A2)	131	0.11	-0.09	0.92	A2 - A3	1.00E+00	ns
	Anaplastic Astrocytoma (A3)	120	0.23	0.03	1.08	GBM - A3	1.00E+00	ns
	Glioblastoma	225	0.26	-0.07	1.2	GBM - A2	1.00E+00	ns

Sd = standard deviation, ns = no significance

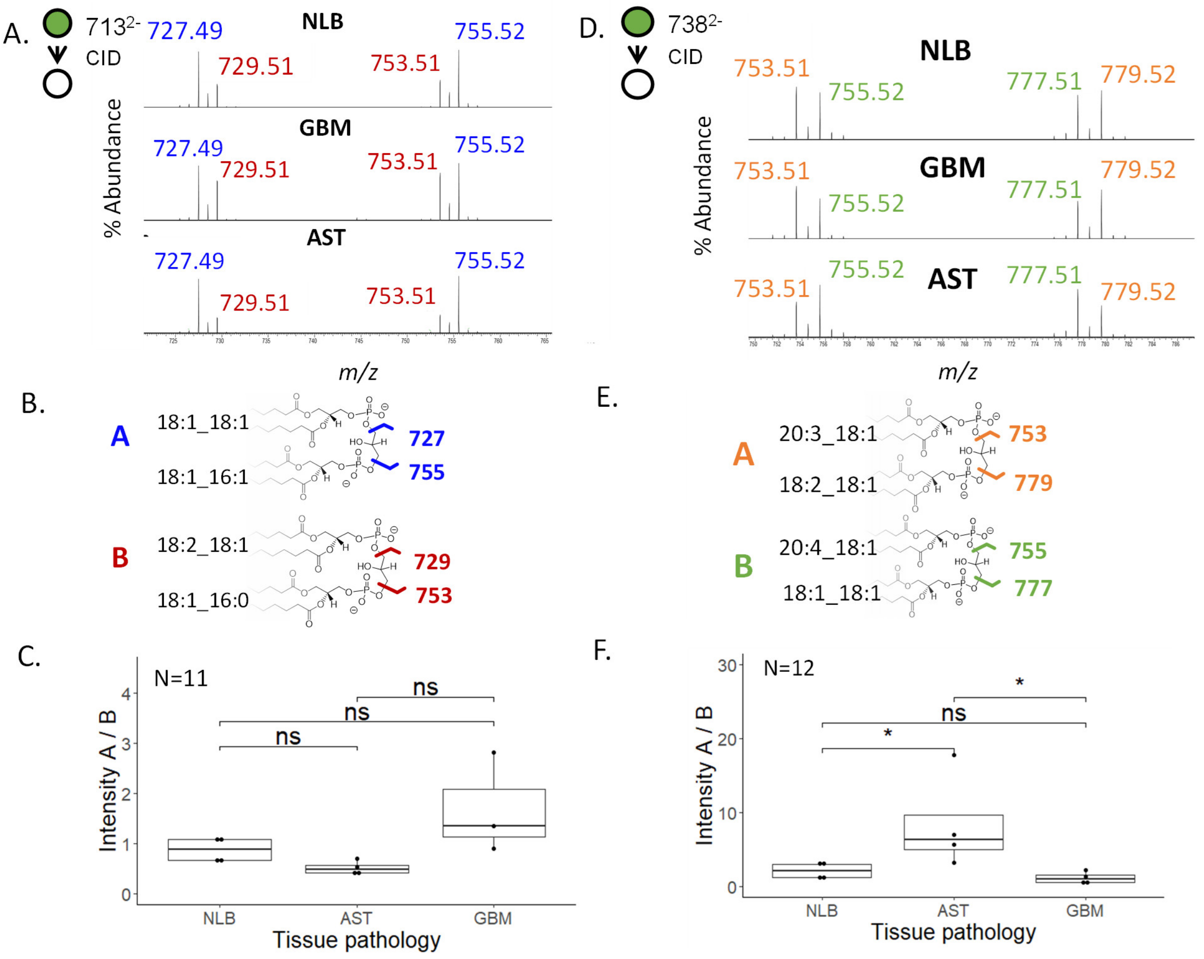

Supplementary Figure S7. A) Representative fragmentation pattern of CID-MS analysis of *m/z* 713 B) fragmentation maps showing PA losses from two prominent isomers A = CL (18:1_18:1_18:1_16:1) and B = CL (18:2_18:1_18:1_16:1) C. boxplots showing averaged intensity of A / B diagnostic ions for each tissue type (N =11). C) representative fragmentation pattern of CID-MS analysis of *m/z* 738 B) fragmentation maps showing PA losses from two prominent isomers A = CL(20:3_18:1_18:2_16:1) and B = CL(20:4_18:1_18:1_18:1) C. boxplots showing averaged intensity of A / B diagnostic ions for each tissue type (N =12). Wilcoxon, *P ≤ 0.05, ns = no significant difference.

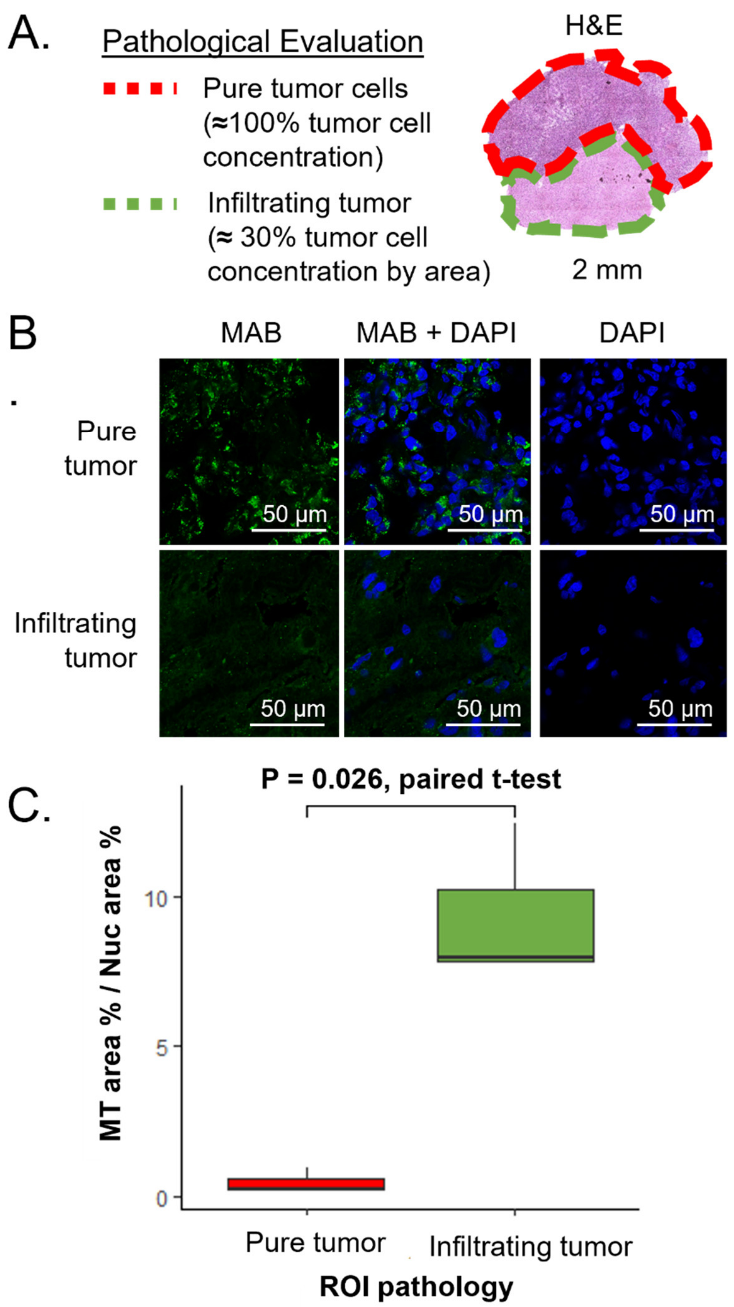

Supplementary Figure S8. A) Pathological evaluation, B) Confocal images for pure tumor and infiltrating tumor regions of a GBM tissue sample (N = 3 ROI for each histological region) stained with anti-mitochondrial antibody (green), DAPI nuclear stain (blue) Scale bar = 50 μm. C) Area % mitochondrial stain versus nuclear stain of distinct histological regions (N = 3 ROI per pathological region) from patient tumor 60 (Paired t-test, P = 0.026).
